# Caveats on Using Firth's Penalization in the Model‐Based Regression Standardization for Rare Diseases

**DOI:** 10.1002/sim.70644

**Published:** 2026-06-23

**Authors:** Sotaro Hashibe, Wataru Hongo, Tomohiro Shinozaki

**Affiliations:** ^1^ Statistics and Decision Sciences Japan, R&D Janssen Pharmaceutical K.K. Tokyo Japan; ^2^ Department of Information and Computer Technology, Graduate School of Engineering Tokyo University of Science Tokyo Japan; ^3^ Advanced Quantitative Sciences Japan, Development Japan Novartis Pharma K.K. Tokyo Japan; ^4^ Interfaculty Initiative in Information Studies the University of Tokyo Tokyo Japan; ^5^ Department of Biostatistics, School of Public Health, Graduate School of Medicine the University of Tokyo Tokyo Japan

**Keywords:** bias reduction, causal effect, Firth, model‐based standardization, separation

## Abstract

Model‐based regression standardization, also known as the parametric g‐formula, is widely used to estimate marginal effect measures. However, in rare disease settings, the small number of observed events relative to the number of covariates can lead to (quasi‐)complete separation, resulting in non‐convergent estimates in the regression models. Firth's penalized likelihood estimates are a common solution to this issue, ensuring finite parameter estimates even for separated data. While effective for estimating regression coefficients, Firth's method introduces bias into model‐based regression standardization because of its tendency to shrink the predicted probabilities to 0.5, leading to discrepancies between the predicted and observed event rates. We examined the implications of applying Firth's method to model‐based regression standardization, illustrating its potential bias through an empirical study on surgical site infections (SSI) in orthopedic surgeries. We also proposed two ad hoc corrections (i.e., Firth's logistic regression with intercept correction and added covariate) to mitigate this bias and evaluated these methods via simulation studies, comparing them with propensity score‐based approaches. Finally, we applied the proposed method to assess the association between SSI, a rare disease, and smoking status in a clinical database of orthopedic surgeries.

## 1 Introduction

Model‐based regression standardization [1], also known as parametric g‐formula or g‐computation for fixed exposures [2], is a popular method for the estimation of marginal effect measures in both randomized and observational studies [3, 4]. For example, logistic regression models for binary outcomes provide not only the covariate‐conditional odds ratios by exponentiated regression coefficients but also the marginal “counterfactual” risks by predicted outcomes under exposed and unexposed conditions, from which risk differences and ratios can be derived as marginal effect measures.

In observational studies of rare diseases, we may encounter a small number of events relative to the number of confounding variables which should be adjusted for in the models. In such cases, a problem known as (quasi‐)complete separation may occur, and the nonexistence of the maximum likelihood estimates of the logistic regression models can be problematic [5, 6, 7]. Even if statistical software or packages provide finite estimates in datasets with “separated” outcomes, the obtained values are contingent on the stopping criterion of the iterative computation in each program [8]. Furthermore, the estimated standard errors of the regression parameters increase to near infinity [7, 9]. If such separation matters, then model‐based regression standardization would also provide invalid effect estimates.

A convenient solution to this convergence issue in generalized linear models, including logistic regression models, is to use Firth's penalized likelihood estimates [6, 10]. This approach may yield finite estimates, even when the maximum likelihood estimates would become infinite owing to separation [6, 9]. Originally designed to correct for first‐order bias in maximum likelihood estimates [10], Firth's penalization is applicable even in situations with sufficient event counts, regardless of the presence of separation [6, 8, 9, 11, 12, 13]. Hence, it may be attractive to apply Firth's method to the estimation of regression parameters in model‐based standardization estimators in rare disease settings without practical concerns. However, the use of Firth's penalization in model‐based standardization has a problem similar to those previously reported by Puhr et al. [14] regarding biases in the estimation of predicted probabilities. Specifically, Firth's penalized likelihood estimates tend to pull the predicted probabilities toward 0.5, leading to a non‐negligible discrepancy between the predicted and observed event rates when events are extremely rare or common [14]. As model‐based standardization estimators are based on the predicted outcomes under different exposures, Firth's method may inflate rather than reduce bias in rare‐event situations, even if the regression coefficients in the model are accurately estimated. Our study examines the potential biases introduced by using Firth's penalized likelihood for estimating marginal effects, within the framework of a counterfactual model in causal inference, thereby underscoring the novelty of this research.

In this study, we illustrate the possible bias using Firth's method for model‐based regression standardization estimators, which has received little attention in the literature. In Section 2, we introduce model‐based regression standardization estimators and Firth's method to estimate the underlying logistic regression models. Section 3 compares regression standardization estimators with or without following Firth's penalization, with illustration using stratified data from a study of surgical site infection (SSI) in orthopedic surgeries. This section also presents two *ad hoc* methods of Firth's penalized estimates that correct the predicted probabilities such that their average equals the observed event rate in each exposure group. These regression standardization methods are evaluated along with propensity score‐based methods using simulation studies in Section 4 in terms of bias and convergence rates. Finally, in Section 5, the association of SSI (which is known as a rare disease) and smoking (a known risk factor for the infection) was analyzed in the database of orthopedic surgeries.

## 2 Regression Standardization Estimators Following Firth's Penalization

### 2.1 Notation and Assumptions

Let $Y_{i}$ be the outcome (1: event occurred, 0: event did not occur), $Z_{i}$ be the exposure (1: exposed, 0: unexposed), and $L_{i}$ be the set of confounding variables for patient i (i=1,…,n). Using a potential outcome $Y_{i}(z)$ under exposure $Z_{i}=z$ with the *causal consistency* assumption, that is, $Y_{i}=Y_{i}(z)$ if $Z_{i}=z$ [15], the marginal causal effect can be defined as the contrast of the potential outcome means

(1) E[Y(1)]−E[Y(0)].

Hereafter, we omit the subscript i if it is unnecessary. This marginal “mean” effect (1) is identifiable from the probability distribution of observed data ($Y_{i},Z_{i},L_{i}$) if the following two assumptions are additionally met [15]: the *mean conditional exchangeability*, i.e., E[Y(z)|Z=z,L]=E[Y(z)|L], and the *conditional positivity*, i.e., 0<P(Z=z|L), across possible support of L and for z=0,1. Under these assumptions, we obtain the following identification formula:

(2) $$E[Y(1)]-E[Y(0)]=\int R(1,l)f_{L}(l)dl-\int R(0,l)f_{L}(l)dl,$$

where R(z,l)=E[Y|Z=z,L=l] is regression of Y on (Z,L) evaluated at (z,l), and $f_{L}(l)$ is the marginal density of L at l.

### 2.2 Model‐Based Regression Standardization Estimators With and Without Penalized Likelihood

To estimate the identification formula (2) from the observed data, we may substitute $f_{L}(l)$ with the empirical distribution (i.e., the probability distribution that places probability 1/n at each observed value $L_{i}$) and R(z,l) with the estimates of the parametric regression models, including the unknown parameters β. The model‐based regression standardization estimator [1], or the parametric g‐formula for a fixed exposure effect [2], is given by

(3) $$\hat{E}[Y(1)]-\hat{E}[Y(0)]=\frac{1}{n}\overset{n}{\underset{i=1}{\sum }}R(1,L_{i};\hat{\beta })-\frac{1}{n}\overset{n}{\underset{i=1}{\sum }}R(0,L_{i};\hat{\beta }),$$

where $R(z,L_{i};\hat{\beta })$ is the outcome predicted from the estimated parametric model for patient i by replacing the exposure status $Z_{i}$ with z=0,1.

The popular parametric model for the binary outcome $Y_{i}$ is a logistic regression model. For example,

$$\mathrm{logit}R(z,l;\beta )=\beta_{0}+\beta_{1}z+\beta_{2}^{⊤}l+\beta_{3}^{⊤}zl,$$

where logitr=log{r/(1−r)} for r∈(0,1). We typically estimate the parameters $\beta ={\left(\beta_{0},\beta_{1},\beta_{2}^{⊤},\beta_{3}^{⊤}\right)}^{⊤}$ by the maximum likelihood, which is equivalent to solving the following score equations:

(4) $$\overset{n}{\underset{i=1}{\sum }}\left\{Y_{i}-R(Z_{i},L_{i};\beta )\right\}X_{i}=0,$$

where $X_{i}={\left(1,Z_{i},L_{i}^{⊤},Z_{i}L_{i}^{⊤}\right)}^{⊤}$ is the shorthand notation for regressors in the logistic model. Using the resulting maximum likelihood estimator ${\hat{\beta }}_{\text{ML}}$, the model‐based standardization estimator can be expressed as follows:

(5) $$\begin{array}{ll} & \frac{1}{n}\overset{n}{\underset{i=1}{\sum }}R(1,L_{i};{\hat{\beta }}_{\text{ML}})-\frac{1}{n}\overset{n}{\underset{i=1}{\sum }}R(0,L_{i};{\hat{\beta }}_{\text{ML}})\\ & \;=\frac{1}{n}\overset{n}{\underset{i=1}{\sum }}\mathrm{expit}\left({\hat{\beta }}_{0,\text{ML}}+{\hat{\beta }}_{1,\text{ML}}+{\hat{\beta }}_{2,\text{ML}}^{⊤}L_{i}+{\hat{\beta }}_{3,\text{ML}}^{⊤}L_{i}\right)\\ & \;-\frac{1}{n}\overset{n}{\underset{i=1}{\sum }}\mathrm{expit}\left({\hat{\beta }}_{0,\text{ML}}+{\hat{\beta }}_{2,\text{ML}}^{⊤}L_{i}\right), \end{array}$$

where $\mathrm{expit}(u)=e^{u}/(1+e^{u})$ is the inverse function of the logit function.

When the number of events is extremely small or large, the maximum likelihood estimates ${\hat{\beta }}_{\text{ML}}$ would suffer from sparse data bias or even cannot be obtained because of the (quasi‐)complete separation [6, 8, 9, 11, 12, 13]. In such cases, the standardization estimators would also become invalid. Firth's method circumvents such a difficulty by penalizing the likelihood function of logistic models, corresponding to solving the following “penalized” score equations [14]:

(6) $$\overset{n}{\underset{i=1}{\sum }}\left\{Y_{i}-R(Z_{i},L_{i};\beta )+h_{i}\left(\frac{1}{2}-R(Z_{i},L_{i};\beta )\right)\right\}X_{i}=0,$$

where $h_{i}=h(X_{i},\beta )$ represents the i‐th diagonal element of the “hat” matrix $H_{\beta }$ such that $H_{{\hat{\beta }}_{\text{FML}}}{\left(Y_{1},\ldots ,Y_{n}\right)}^{⊤}={\left(R(Z_{1},L_{1};{\hat{\beta }}_{\text{FML}}),\ldots ,R(Z_{n},L_{n};{\hat{\beta }}_{\text{FML}})\right)}^{⊤}$, producing predicted outcomes at the Firth's maximum penalized likelihood estimates satisfying the equations (6).

The regression standardization estimators are obtained simply by replacing ${\hat{\beta }}_{\text{ML}}$ with ${\hat{\beta }}_{\text{FML}}$ in (5). Although there is minimal bias in ${\hat{\beta }}_{\text{FML}}$ for β in a correctly specified model [16], the standardization estimators based on Firth's penalized likelihood estimates may be biased owing to the known property of predicted outcome probabilities $R(Z_{i},L_{i};{\hat{\beta }}_{\text{FML}})$ [14]. The predicted probabilities tend to be pulled toward 0.5, as described in the next section.

## 3 Discrepancy Between Standardization Estimators Following the Standard and Penalized Maximum Likelihood Estimation

### 3.1 Systematic Change in Predicted Probabilities Due to Firth's Penalization

Unlike the standard maximum likelihood method, the average predicted probability using Firth's method is not equal to the observed event rate [14]. Namely, (1) the *average* of the predicted probabilities $R(Z_{i},L_{i};{\hat{\beta }}_{\text{FML}})$ is in between the average of predicted probabilities $R(Z_{i},L_{i};{\hat{\beta }}_{\text{ML}})$ (which is equal to the event rate in a sample) and 0.5 and (2) *each* predicted probability $R(Z_{i},L_{i};{\hat{\beta }}_{\text{FML}})$ tends to be pulled to 0.5 from $R(Z_{i},L_{i};{\hat{\beta }}_{\text{ML}})$ [14, 17]. To explain these results, we rewrite the penalized score equations (6) as follows:

(7) $$\begin{array}{cc} & \overset{n}{\underset{i=1}{\sum }}\left\{\left(Y_{i}-R(Z_{i},L_{i};\beta )\right)\right.\\ & \;\left.+\frac{h_{i}}{2}\left(1-R(Z_{i},L_{i};\beta )\right)+\frac{h_{i}}{2}\left(0-R(Z_{i},L_{i};\beta )\right)\right\}X_{i}=0. \end{array}$$

This indicates that equations (6) augment the standard score equations (4) by introducing two artificial “copies” $\left(y,Z_{i},L_{i}\right)$ of the data $\left(Y_{i},Z_{i},L_{i}\right)$, where the outcomes are replaced with y=1 and y=0, and weighted by $h_{i}/2$. Note that $0<h_{i}\leq 1$ such that $\sum_{i=1}^{n}h_{i}=\dim(\beta )$ if the design matrix of a logistic model is full‐rank [14, 17]. Hence, it is clear that the event rate in this “augmented” dataset, which is equal to average $R(Z_{i},L_{i};{\hat{\beta }}_{\text{FML}})$, is closer to 0.5 than the event rate in the original dataset, which is equal to average $R(Z_{i},L_{i};{\hat{\beta }}_{\text{ML}})$. Moreover, the above expression also provides insight that each predicted probability $R(Z_{i},L_{i};{\hat{\beta }}_{\text{FML}})$ tends to be pulled toward 0.5 because one event (y=1) and one non‐event (y=0) with the same $\left(Z_{i},L_{i}\right)$ are augmented with the same weight $h_{i}/2$ in the penalized equations (7). In fact, as illustrated in the next subsection, a simple condition exists that ensures that each $R(Z_{i},L_{i};{\hat{\beta }}_{\text{FML}})$ is closer to 0.5 than $R(Z_{i},L_{i};{\hat{\beta }}_{\text{ML}})$ if saturated regression models are used.

Hereafter, we assume the event rates to be much smaller than 0.5 in both exposure groups. Furthermore, because $X_{i}$ includes an exposure indicator $Z_{i}$, the relationship above for the average probabilities remains true for each exposure group, leading to the following inequalities:

$$\begin{array}{ll} \overset{n}{\underset{i=1}{\sum }}Z_{i}R(Z_{i},L_{i};{\hat{\beta }}_{\text{ML}}) & <\overset{n}{\underset{i=1}{\sum }}Z_{i}R(Z_{i},L_{i};{\hat{\beta }}_{\text{FML}})<0.5,\\ \overset{n}{\underset{i=1}{\sum }}(1-Z_{i})R(Z_{i},L_{i};{\hat{\beta }}_{\text{ML}}) & <\overset{n}{\underset{i=1}{\sum }}(1-Z_{i})R(Z_{i},L_{i};{\hat{\beta }}_{\text{FML}})<0.5. \end{array}$$

The standardization estimators also rely on the predicted outcomes under “opposite” exposure status, that is, $R(1-Z_{i},L_{i};\hat{\beta })$. The standardization estimator $\hat{E}[Y(z)]$ based on Firth's penalized maximum likelihood estimator is

$$\begin{array}{cc} & {\hat{E}}_{\text{FML}}[Y(1)]=\frac{1}{n}\overset{n}{\underset{i=1}{\sum }}R(1,L_{i};{\hat{\beta }}_{\text{FML}})\\ & \;=\frac{1}{n}\overset{n}{\underset{i=1}{\sum }}\left\{Z_{i}R(Z_{i},L_{i};{\hat{\beta }}_{\text{FML}})+(1-Z_{i})R(1-Z_{i},L_{i};{\hat{\beta }}_{\text{FML}})\right\}\\ & {\hat{E}}_{\text{FML}}[Y(0)]=\frac{1}{n}\overset{n}{\underset{i=1}{\sum }}R(0,L_{i};{\hat{\beta }}_{\text{FML}})\\ & \;=\frac{1}{n}\overset{n}{\underset{i=1}{\sum }}\left\{\left(1-Z_{i})R(Z_{i},L_{i};{\hat{\beta }}_{\text{FML}})+Z_{i}R(1-Z_{i},L_{i};{\hat{\beta }}_{\text{FML}}\right)\right\} \end{array}$$

The first terms in the summation of RHSs of these equations are larger than their counterparts based on the maximum likelihood estimates ${\hat{\beta }}_{\text{ML}}$. Unfortunately, however, we do not have further general results regarding the relationship between standardization estimators with and without Firth's penalization because the average “counterfactual” predictions (i.e., the second terms) may or may not become greater by Firth's penalization than that from maximum likelihood estimates in each exposure group.

However, as the next subsection demonstrates, we can derive a simple sufficient condition for $R(z,l;{\hat{\beta }}_{\text{FML}})$ to be larger than $R(z,l;{\hat{\beta }}_{\text{ML}})$ for the given combinations of (z,l) if we employ saturated logistic regression models. We defer the quantitative evaluation in more practical settings with parametric models, as well as the examination of whether the discrepancy between these standardization estimators leads to a bias, to the next section.

### 3.2 Illustration Using Stratified Data

Table 1 represents the data from the Society for Orthopedic Surgical Site Infection (OSSI), which consists of seven hospitals in the metropolitan area of Japan [18]. Briefly, the Society for OSSI prospectively collected the preoperative, intraoperative, and postoperative variables of consecutive patients undergoing orthopedic surgery in a clean surgical environment between 2013 and 2016. Overall, 10 943 eligible patients were enrolled, and 148 variables were measured.

**TABLE 1:** Smoking–surgical site infection (SSI) association stratified by American Society of Anesthesiologists (ASA) classification among patients undergoing arthropathy surgeries in the data obtained from the Society of OSSI (n=2717).

		ASA classification ≥3 (L=1)		ASA classification ≤2 (L=0)	
		SSI (Y=1)	No SSI (Y=0)	SSI (Y=1)	No SSI (Y=0)
Smoking (Z=1)		1	5	2	187
No smoking (Z=0)		1	107	11	2403

Prognostic factors for SSI events include age, sex, American Society of Anesthesiologists (ASA) classification of severity, and diabetes, with smoking being particularly important [19]. In the present study, we examined the association between smoking habits (Z=1: smoker, 0: non‐smoker) as an exposure and incidence within 30 days after surgery (Y=1: occurrence, 0: no occurrence) as an outcome, adjusting for ASA classification (L=1: ≥3, 0: ≤2) as a confounder. Postoperative complications, including SSI, are generally more common in smokers than in nonsmokers, and a previous report revealed that perioperative smoking cessation reduces SSIs [20]. In this database, most non‐arthritic surgeries required perioperative hospitalization, which led patients to stop smoking. Hence, Table 1 presents only n=2717 arthropathic surgeries as an illustrative example as these patients were not necessarily hospitalized before surgery.

We modeled the conditional probabilities for Y=1 in the stratified data in Table 1 using the saturated logistic model:

$$\mathrm{logit}P(Y=1|Z=z,L=l)=\beta_{0}+\beta_{1}z+\beta_{2}l+\beta_{3}zl.$$

Using the estimated conditional probabilities from the estimates of the saturated model, the maximum likelihood–based standardization estimates (3) yielded:

$$\begin{array}{cc} {\hat{E}}_{\text{ML}}[Y(1)] & =\frac{1}{6}\left(\frac{114}{2717}\right)+\frac{2}{189}\left(\frac{2603}{2717}\right)=1.713\%,\\ {\hat{E}}_{\text{ML}}[Y(0)] & =\frac{1}{108}\left(\frac{114}{2717}\right)+\frac{11}{2414}\left(\frac{2603}{2717}\right)=0.475\%. \end{array}$$

Although Firth's penalization can be applied to the saturated logistic model using statistical software, it is mathematically equivalent to obtaining the maximum likelihood estimates from a contingency table with pseudo‐counts of +0.5 added to each cell of Table 1 [6]. As Firth's penalization affects only the estimation of conditional probabilities R(z,l)=P(Y=1|Z=z,L=l), the resulting standardization estimates are as follows:

$$\begin{array}{cc} {\hat{E}}_{\text{FML}}[Y(1)] & =\frac{1.5}{7}\left(\frac{114}{2717}\right)+\frac{2.5}{190}\left(\frac{2603}{2717}\right)=2.160\%,\\ {\hat{E}}_{\text{FML}}[Y(0)] & =\frac{1.5}{109}\left(\frac{114}{2717}\right)+\frac{11.5}{2415}\left(\frac{2603}{2717}\right)=0.514\%. \end{array}$$

Hence, ${\hat{E}}_{\text{FML}}[Y(z)]>{\hat{E}}_{\text{ML}}[Y(z)]$ for both z=0,1 in this dataset.

There is a sufficient condition for Firth's penalization to inflate the maximum likelihood‐based standardization estimates using saturated regression models. As demonstrated above, the predicted outcome probability $R(z,l;{\hat{\beta }}_{\text{ML}})$ from the saturated regression models is equal to the exposure z‐specific observed event rates in confounder stratum L=l. We also demonstrated that Firth's penalization in the likelihood of the saturated regression model was equivalent to the maximum likelihood estimation applied to the augmented data, where all stratified cell counts are increased by 0.5. Let $N_{z,k}$ denote the number of patients exposed to Z=z in the confounder stratum $L=l_{k}$ (k=1,…,K; for example, in Table 1, K=2 such that $l_{1}=1$ and $l_{2}=0$), of which $A_{z,k}$ patients develop events. The predicted outcome under Z=z following Firth's penalization $R(z,l_{k};{\hat{\beta }}_{\text{FML}})$ is larger than the maximum likelihood prediction, $R(z,l_{k};{\hat{\beta }}_{\text{ML}})$ if and only if $(A_{z,k}+0.5)/(N_{z,k}+1)-A_{z,k}/N_{z,k}>0$, or $A_{z,k}<N_{z,k}-A_{z,k}$. In other words, this inequality suggests that the event rate in the exposure Z=z is lower than 0.5, in stratum $l_{k}$. Hence, ${\hat{E}}_{\text{FML}}[Y(z)]$ is necessarily greater than ${\hat{E}}_{\text{ML}}[Y(z)]$ if the observed event rates in the exposure Z=z are smaller than 0.5 across all strata $l_{1},\ldots ,l_{K}$.

### 3.3 Ad Hoc Modiications of Standardization Estimators

To address the overestimation (relative to the standard maximum likelihood) in regression standardization estimators following Firth's maximum penalized likelihood, we apply two modified algorithms proposed in the context of prediction modeling: Firth's logistic regression with intercept correction (FLIC) and added covariate (FLAC) methods [14]. Both methods are implemented using the R package logistf (https://cran.r‐project.org/web/packages/logistf/index.html) [21]. The following simple modifications match the average predicted probabilities with the observed event proportions:

- FLIC refits the logistic model with only an intercept term by including a linear predictor (i.e., logit predicted outcome) as an offset by standard maximum likelihood. That is, we update the predicted values by reestimating $\beta_{\text{FLIC}}$ in $\mathrm{logit}P(Y=1|Z,L)=\beta_{\text{FLIC}}+{\hat{\beta }}_{0,\text{FML}}+{\hat{\beta }}_{1,\text{FML}}Z+{\hat{\beta }}_{2,\text{FML}}^{⊤}L+{\hat{\beta }}_{3,\text{FML}}^{⊤}ZL=\beta_{\text{FLIC}}+\mathrm{logit}R(Z,L;{\hat{\beta }}_{\text{FML}})$.
- FLAC modifies the penalized score equations (7) by the following algorithm: (1) replicate each data $\left(Y_{i},Z_{i},L_{i}\right)$ by 3 copies (sub‐indexed by j=1,2,3); (2) replace outcome $Y_{i,2}$ by 1 and $Y_{i,3}$ by 0 (keep $Y_{i,1}=Y_{i}$ as original data); (3) define a dummy variable $G_{i,j}$ such that $G_{i,1}=0$ and $G_{i,2}=G_{i,3}=1$; (4) add weights $W_{i,1}=1$ and $W_{i,2}=W_{i,3}=h_{i}/2$, where $h_{i}$ is obtained from the hat matrix of initial estimation from Firth's method; and (5) fit the logistic model $\mathrm{logit}P(Y=1|Z,L,G)=\beta_{0,\text{FLAC}}+\beta_{1,\text{FLAC}}Z+\beta_{2,\text{FLAC}}^{⊤}L+\beta_{3,\text{FLAC}}^{⊤}ZL+\beta_{4,\text{FLAC}}G$ in augmented datasets by replicated copies (i=1,…,n;j=1,2,3) using weighted maximum likelihood with weights $W_{ij}$.

Both the maximum likelihood fit for FLIC and the (weighted) maximum likelihood fit with an added dummy variable $G_{i,j}$ with weight $W_{i,j}$ in the augmented data for FLAC ensure the equivalence of the average predicted outcome and observed event rates in the original dataset [14]. To apply them to the model‐based regression standardization estimators, we replace $R(z,L_{i};{\hat{\beta }}_{\text{ML}})$ in Equation (5) with the corresponding outcome predictions:

$$\begin{array}{ll} & {\hat{E}}_{\text{FLIC}}[Y(z)]=\frac{1}{n}\overset{n}{\underset{i=1}{\sum }}\mathrm{expit}\{{\hat{\beta }}_{\text{FLIC}}+\mathrm{logit}R(z,L_{i};{\hat{\beta }}_{\text{FML}})\},\\ & {\hat{E}}_{\text{FLAC}}[Y(z)]\\ & \;=\frac{1}{n}\overset{n}{\underset{i=1}{\sum }}\mathrm{expit}({\hat{\beta }}_{0,\text{FLAC}}+{\hat{\beta }}_{1,\text{FLAC}}z+{\hat{\beta }}_{2,\text{FLAC}}^{⊤}L_{i}+{\hat{\beta }}_{3,\text{FLAC}}^{⊤}zL_{i}). \end{array}$$

Table 2 depicts the results of the regression standardization based on the saturated logistic model presented in Table 1. All Firth's estimates were obtained from logistic model fits using the R package logistf. While applying Firth's penalization can result in higher standardization estimates than those obtained using maximum likelihood estimation, FLIC and FLAC can mitigate this overestimation even when Firth's penalization is applied. However, in this analysis, we did not evaluate whether the deviation in Firth's method (2nd row) from the others should be considered a bias; this issue is addressed in the next section using simple simulations.

**TABLE 2:** Estimates of model‐based standardization in the stratified data from Table 1 using saturated logistic models with different fitting methods.

	$\hat{E}[Y(1)]$	$\hat{E}[Y(0)]$	Difference	Ratio
Maximum‐likelihood estimation	1.713%	0.475%	1.238%	3.60
Firth's method	2.16%	0.514%	1.646%	4.20
FLIC	1.949%	0.459%	1.490%	4.24
FLAC	1.471%	0.488%	0.982%	3.01

## 4 A Simulation Study

### 4.1 Data Generation

We evaluated the bias of model‐based regression standardization using distinct estimates $\hat{\beta }$ (i.e., maximum likelihood, Firth's penalized maximum likelihood, FLIC, and FLAC) for estimating the marginal causal risks E[Y(z)] (z=0,1), marginal risk difference E[Y(1)]−E[Y(0)], and the log marginal risk ratio log{E[Y(1)]/E[Y(0)]} through a simple simulation study, assuming situations with rare events and the possibility of separation occurring. Each simulation was repeated 1000 times. The simulations were performed using R version 4.2.2.

For data i=1,…,n=(500,2500) in each repetition, we generated 10‐dimensional latent variables from a multivariate normal distribution with mean vector fixed at zero and covariance matrix ∑, where diagonal elements were set to 1 and off‐diagonal elements were set to ρ∈{0.5,0.25,0}. These latent variables were then converted into binary variables $L_{i}=(L_{i1},\ldots ,L_{i10})$ using an indicator function for values greater than zero. Additional details of the data‐generating parameters for the exposure and outcome models are provided in the Supporting Information, with the full set of parameters listed in Supporting Information Tables 1 and 2.

(8) $$\mathrm{logit}P(Z=1|L)=\alpha_{0}+\overset{10}{\underset{k=1}{\sum }}\alpha_{k}L_{k}$$

and outcome follows the logistic model

(9) $$\mathrm{logit}P(Y=1|Z,L)=\beta_{0}+\overset{10}{\underset{k=1}{\sum }}\beta_{k}L_{k}+\beta_{Z}Z,$$

where $\beta_{Z}$ was set as log(0.8).

Exposure probability was set to 10%, and the event rates were 20%, 10%, 5%, 3%, 1%, and 0.5%. For each event rate, the frequency of separation was controlled by the correlation between the latent normal variables behind the confounders (0, 0.25, and 0.5). We manipulated the intercepts $\alpha_{0}$ and $\beta_{0}$ to achieve the desired event rates.

As the analytical representation of true marginal risks and their contrasts (difference and log ratio) is not feasible, we numerically approximated the targeted values by directly generating $Y_{i}(1)$ and $Y_{i}(0)$ with a sample size of n=10,000,000.

While our simulations were conducted under conditions that avoided separation in the propensity score model, we emphasize that the absence of separation does not a sufficient condition for satisfying the positivity assumption and/or ensuring adequate covariate overlap between the groups. Positivity violations can be either deterministic (precluding nonparametric *identifiability*) or stochastic (affecting finite‐sample *estimability*); separation in a propensity score model can be viewed as a manifestation of a lack of estimability, which may or may not result from (stochastic or deterministic) positivity violation [22]. Hence, in practical applications, the (deterministic) positivity assumption required for nonparametric identifiability should be assessed based on subject‐matter knowledge, and potential near‐violations of positivity should be diagnosed beyond checking for separation to ensure valid inference. To this end, the overlap of the covariates and estimated propensity score distributions between exposure groups should be examined visually and statistically [23]. Researchers should also be alert to sparsity in covariate strata, where random positivity violations can occur because of small sample sizes or high‐dimensional data. In practice, such sparsity may be identified by examining contingency tables of key categorical covariates, where empty or near‐empty cells may indicate limited support. In such cases, researchers may consider simple remedies such as collapsing categories or treating them as continuous covariates in regression and propensity score models. When inverse probability weights are used, the distribution of the resulting weights should also be examined. For example, estimated weights with very extreme values may indicate nonpositivity or model misspecification [24]. When extreme weights affect the stability of the analysis, *ad hoc* methods such as weight truncation may offer a better tradeoff between bias and precision. In our simulation, these issues were mitigated by design, but such diagnostics are essential when applying confounding‐adjustment methods to real‐world data.

### 4.2 Evaluated Methods and Performance Measures

Six estimation methods were compared. In addition to the maximum likelihood with or without the Firth method, including the FLIC and FLAC modifications, two propensity score‐based estimators were employed to reduce the number of adjustment variables in the outcome regression models. The first is the model‐based regression standardization estimator based on the logistic regression model, which adjusts for the estimated propensity scores instead of confounders $L_{i}$. The second method is an inverse probability weighted (IPW) estimator. The maximum‐likelihood estimates of propensity scores were obtained through a logistic model for e(L)=P(Z=1|L), namely $e\left(L_{i};{\hat{\alpha }}_{\text{ML}}\right)=\mathrm{expit}({\hat{\alpha }}_{0,\text{ML}}+{\hat{\alpha }}_{1,\text{ML}}^{⊤}L_{i})$. Both propensity score‐based methods have been demonstrated to circumvent instability owing to sparse data problems in rare disease settings [25, 26, 27, 28, 29, 30].

Separation was detected by monitoring the standard error estimates of the parameters as established in [31]. We used the R package detectseparation to determine whether separation occurs by specifying method = “detect_separation” in the glm function [32]. In addition, if no event occurs in either exposure group, maximum likelihood estimates without Firth's penalization and propensity score‐based estimates cannot be obtained from such datasets. We recorded them as “Not Applicable (NA)” and did not include them in the summary measures.

We estimated the marginal risks E[Y(1)] and E[Y(0)], along with their differences and log‐transformed ratios. The asymptotic standard errors were estimated using multivariate delta methods (for regression standardization estimators) or the (conservative) sandwich estimator (for IPW estimators). The estimates were evaluated using bias (i.e., estimated − true effect), relative bias (i.e., bias divided by true values in Supporting Information Table 3), Monte Carlo standard error (MCSE), mean estimated standard error (MESE), and coverage proportion of the 95% confidence interval. To assess the reliability of the simulation results, we evaluated Monte Carlo standard errors (MCSE) and mean estimated standard errors (MESE). Across all scenarios, MCSE values were small relative to the estimated effects, and MESE closely matched the empirical standard deviations, indicating that the stable and reliable inference. As the true effect is substantially small, the relative bias is reported in the main text to clarify the extent of bias against the true effect, whereas the bias itself is reported in the Supporting Information. The R code for the simulation experiments is provided in the Supporting Information.

### 4.3 Results

Among 1000 replications, separation occurred only in the scenario with an event rate of 0.005 under a sample size of 2500; the separation rates were 24% (between‐covariate correlation: 0), 83% (correlation: 0.25), and 90% (correlation: 0.5), resulting in “NA” values for maximum‐likelihood estimation without Firth's penalization (see Supporting Information Table 2). For propensity score‐based estimators, we excluded replications in which at least one cell in the (Y,Z) contingency table had zero counts (see Supporting Information Table 2). Exclusions were negligible in most scenarios but increased sharply when events were rare. At an event rate of 0.5%, exclusions ranged from 0.0% to 49.3% of replications (0–493 out of 1000), depending on the sample size and the between‐covariate correlation. Accordingly, PS‐based results were summarized using a scenario‐specific denominator of 1000−m, where m denotes the number of excluded replications.

**FIGURE 1:**
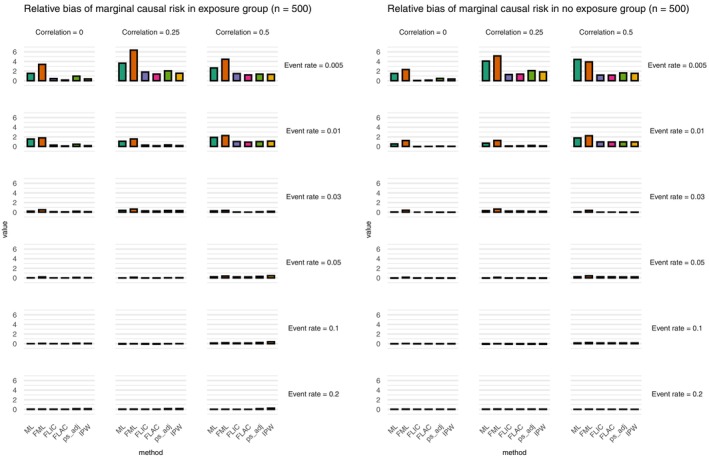
Relative bias for E[Y(1)] and E[Y(0)] (n=500). The compared methods are regression standardization with maximum likelihood estimates (ML), ML not adjusted for confounding variables (Unadj), regression standardization following Firth's method (FML), regression standardization following Firth's method with corresponding modification (FLIC, FLAC), regression standardization with propensity score‐adjusted model (PS‐adj), and inverse probability weighted estimates (IPW).

**FIGURE 2:**
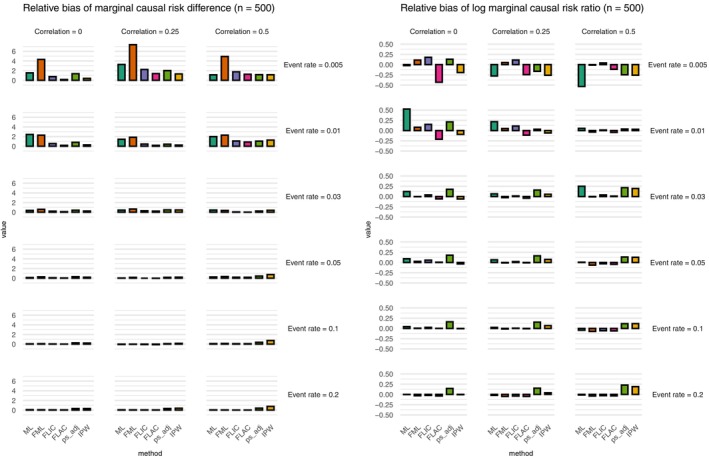
Relative bias for E[Y(1)]−E[Y(0)] and log(E[Y(1)])−log(E[Y(0)]) (n=500). The compared methods are ML, Unadj, FML, FLIC, FLAC, PS‐adj, and IPW.

**FIGURE 3:**
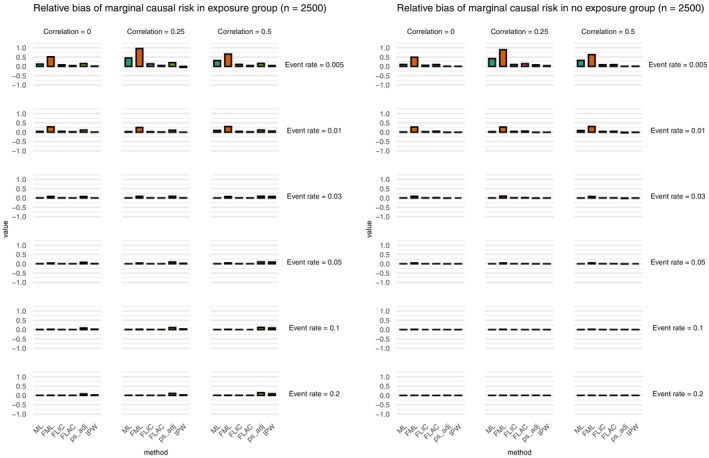
Relative bias for E[Y(1)] and E[Y(0)] (n=2500). The compared methods are ML, Unadj, FML, FLIC, FLAC, PS‐adj, and IPW.

**FIGURE 4:**
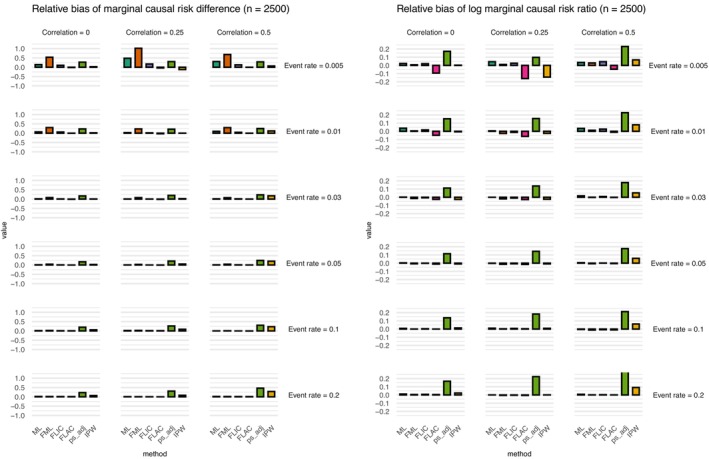
Relative bias for E[Y(1)]−E[Y(0)] and log(E[Y(1)])−log(E[Y(0)]) (n=2500). The compared methods are ML, Unadj, FML, FLIC, FLAC, PS‐adj, and IPW.

Figures 1, 2, 3, 4 shows the relative bias of each method, and the relative bias of the unadjusted risk estimates is shown in Supporting Information Figure 1. As expected, the relative bias was larger at smaller event rates for the regression standardization methods, especially for the maximum likelihood and Firth's estimates. This trend was not observed in the propensity score‐based methods. Increasing the correlation between covariates did not necessarily increase the bias for all methods.

When we focused on rare‐event scenarios prone to separation (event rate 0.005 with nonzero between‐covariate correlation), the relative bias of E[Y(z)] at n=2500 ranged from 10% to 45% for maximum likelihood estimation and from 50% to 95% for Firth's method across z=0 and z=1; FLIC and FLAC exhibited smaller ranges. A similar pattern was observed for the marginal risk differences. At n=500, the qualitative patterns were consistent with those at n=2500; the relative bias increased as the event rate decreased for regression‐standardization methods, particularly for maximum likelihood estimation and Firth's method, whereas FLIC and FLAC showed comparatively smaller biases. The ranges were wider owing to more frequent separation and, for propensity score‐based methods, exclusions based on zero cells in the (Y,Z) table; however, the overall conclusions regarding relative performance and coverage remained unchanged.

Relative biases in the propensity score‐based methods were generally larger than those in the four multivariate‐adjusted regression model‐based standardization methods. Specifically, propensity score‐adjusted regression standardization exhibited a non‐negligible bias, which may be explained by model misspecification due to including propensity scores rather than confounders in the outcome regression models. The IPW estimator provides relatively large biases, especially under large correlations between confounders. This is possible because correlation may have caused instability in the propensity score estimates, which is known to result in finite‐sample bias in weighted estimators. In contrast, propensity score‐based methods demonstrated no consistent monotonic trends across event rates. For propensity score‐based estimators, we excluded replications in which at least one (Y,Z) cell had zero counts, according to our pre‐specified rule (see Supporting Information Table 2). As a result, summaries are reported using a scenario‐specific denominator of 1000−m, where m denotes the number of excluded replications. In rare‐event settings (event rate 0.005), such exclusions ranged from 0.0% to 49.3% of replications, depending on the sample size and the correlation structure.

The MCSE and MESE (see Supporting Information Figures 2 and 3) were comparable between the methods across all scenarios. Across all scenarios and estimators, the ratios of MCSE to MESE were generally close to 1 (ranging approximately from 0.9 to 1.5), indicating that the estimated standard errors were reasonably well calibrated. No systematic pattern of severe miscalibration was observed. The ratios of MCSE to MESE are provided in Supporting Information Tables 4 to 7 for completeness. We defined “approximately nominal” 95% coverage as 95%±2×MCSE (with 1000 replications, MCSE is approximately 0.7 percentage points, corresponding to a range of 93.6%–96.4%). Under this criterion, the 95% coverage rates of all methods, except for the maximum likelihood estimator, were nominal in most scenarios (Supporting Information Figure 3). The undercoverage of the maximum likelihood estimator was concentrated in settings with frequent separation, which reduced the yield of finite estimates (separation rates of up to 90% in the rare‐event scenarios).

## 5 Analysis of the Data From the Society of OSSI

We analyzed the data from the Society for OSSI (Section 3.2) in a more realistic manner by adjusting for multiple confounders using parametric models. Note that maximum likelihood estimates under the absence of events in at least one level of each categorical covariate may lead to non‐convergent estimates or produce “NA” results dependent on the “stopping” criteria of a fitting procedure used in statistical packages. We used the glm function in the R package to fit the models using maximum likelihood.

The outcome of interest was the occurrence of SSI within 30 days of surgery, with smoking as the exposure variable. We estimated the marginal causal risks E[Y(1)] and E[Y(0)] and their difference and ratio using the methods compared in the simulation experiments. Based on a prior publication from the OSSI study [18], we adjusted for the two distinct sets of covariates as linear terms. Adjustment Set 1 included age category (20–29, 30–39,…, 90–99, ≥100), gender, ASA classification (≤ 2, ≥ 3), and presence or absence of diabetes. Adjustment Set 2 additionally included BMI category (< 25,25–< 30, 30≤), total surgical time (< 60, 60–150, 150≤ min), postoperative drainage duration (none, < 48, 48 h or more), presence of rheumatoid arthritis, timing of prophylactic antibiotics (none, < 24, 24 h or more), highest postoperative blood glucose level (< 200 mg/dL, ≥200 mg/dL, not measured), surgical duration (hours), and blood loss (ml). These variables were selected based on their clinical relevance. Supporting Information Table 8 presents the distribution of variables among smokers and nonsmokers.

Table 3 shows a series of estimates. The results after adjusting for Adjustment Set 1 are similar to those in Table 2, which stratifies the data only by diabetes. Note that parametric modeling by logistic regression with linear terms circumvents the quasi‐complete separation of outcomes by model parameters, which is observed in the stratified data (i.e., the saturated model) in Table 1. With Adjustment Set 2, the maximum likelihood estimates could not be obtained owing to quasi‐complete separation. Firth's penalized likelihood estimates provide larger estimates for both marginal risks, E[Y(1)] and E[Y(0)]. Correcting these estimates using FLIC and FLAC decreases risk by approximately 1/3−1/2. The propensity score‐based IPW estimates provide a slightly lower risk under the exposed E[Y(1)], resulting in a smaller risk ratio than that obtained by the other methods.

**TABLE 3:** Estimates of the marginal risk difference and ratio by adjusting for confounding variables by logistic regression model or propensity score model in arthritic patients from the Society of OSSI data (n=2717).

	Adjustment set 1^a^				Adjustment set 2^a^			
	$\hat{E}[Y(1)]$	$\hat{E}[Y(0)]$	Difference	Ratio	$\hat{E}[Y(1)]$	$\hat{E}[Y(0)]$	Difference	Ratio
	(95% CI)	(95% CI)	(95% CI)	(95% CI)	(95% CI)	(95% CI)	(95% CI)	(95% CI)
ML	0.016	0.005	0.012	3.469	NA	NA	NA	NA
	(0.000, 0.037)^b^	(0.002, 0.007)	(−0.009, 0.032)	(0.886, 13.586)	NA	NA	NA	NA
FML	0.021	0.006	0.015	3.721	0.025	0.008	0.017	3.294
	(0.000, 0.043)^b^	(0.003, 0.008)	(−0.007, 0.037)	(1.115, 12.42)	(0.001, 0.049)	(0.004, 0.011)	(−0.007, 0.041)	(1.132, 9.586)
FLIC	0.017	0.005	0.013	3.733	0.016	0.005	0.011	3.348
	(0.000, 0.038)^b^	(0.002, 0.007)	(−0.008, 0.033)	(0.991, 14.061)	(0.000, 0.035)^b^	(0.002, 0.007)	(−0.009, 0.031)	(0.841, 13.324)
FLAC	0.015	0.005	0.01	3.009	0.013	0.005	0.008	2.622
	(0.000, 0.032)^b^	(0.002, 0.008)	(−0.008, 0.028)	(0.801, 11.308)	(0.000, 0.029)^b^	(0.002, 0.008)	(−0.008, 0.024)	(0.723, 9.513)
PS‐adj	0.018	0.005	0.013	3.806	0.016	0.005	0.011	3.402
	(0.000, 0.04)^b^	(0.002, 0.007)	(−0.009, 0.035)	(0.99, 14.636)	(0.000, 0.036)^b^	(0.002, 0.007)	(−0.009, 0.032)	(0.858, 13.482)
IPW	0.014	0.005	0.01	3.077	0.009	0.005	0.004	1.95
	(0.000, 0.033)^b^	(0.002, 0.007)	(−0.009, 0.029)	(7.47, 12.674)	(0.000, 0.021)^b^	(0.002, 0.007)	(−0.007, 0.016)	(0.494, 7.698)

Abbreviations: CI, confidence interval; FML, Firth's penalized likelihood estimates; IPW, inverse probability weighting; ML, maximum likelihood; NA, not applicable; PS‐adj, propensity score adjustment.

^a^ Refer to the main text for variables contained in Adjustment Sets 1 and 2.

^b^ The confidence intervals were truncated at 0 and 1 for marginal risks.

## 6 Discussion

Using real and simulated datasets, this study points out caveats in the application of Firth's method to regression standardization estimators, which have received little attention in the context of causal inference, and evaluates a simple remedy for them. In particular, Firth's method has a non‐negligible bias when estimating marginal counterfactual risks (and their difference) in situations where outcome separation occurs. These biases can be reduced by *ad hoc* modifications such as FLIC and FLAC.

Our simulations revealed that in situations where outcome separation occurred, the regression standardization estimators following Firth's penalized likelihood estimation of logistic models yielded approximately 50%−−66% relative bias. These biases do not necessarily cancel out when estimating the risk differences or log risk ratios, as they may be introduced at different magnitudes in the exposed and unexposed groups. The biases were mitigated using FLIC and FLAC without inflating the standard error. The confidence intervals were also estimated sufficiently correctly using the typical multivariate delta method by replacing the parameter estimates with those from FLIC or FLAC. We also confirmed that the bias in regression standardization using Firth's method is negligible in situations where separation is absent, and the event proportion is not extremely low. However, we have not yet precisely determined the event‐rate threshold at which the FML begins to exhibit a notable bias. Moreover, various factors, such as exposure prevalence, outcome‐model misspecification, magnitude of the exposure effect, absolute number of events, and events per variable may influence this threshold. Therefore, future investigations should focus on the event‐rate threshold and these influencing factors.

In targeting the marginal log–marginal risk ratio as an effect measure, all regression standardization estimators, including Firth's method, suffer from a relative bias of up to 3%. In rare diseases, marginal risk ratios are approximated by marginal odds ratios, which are further approximated by the conditional odds ratios modeled in logistic regression. As Firth's method was originally proposed to reduce bias in typical maximum likelihood estimates of regression coefficients in logistic models [14, 33], the marginal risk ratio can be estimated in an approximately unbiased manner by estimating an exposure coefficient, which represents the conditional odds ratio, with rare events.

In the primary simulations, we assumed that the regression models were correctly specified. However, additional robustness checks under two model misspecification settings yielded qualitatively similar conclusions. Because correctly specifying all working models is challenging in practical data analyses, the sensitivity of the methods to model misspecification remains an important topic for applied use. In practice, however, the problem of quasi‐complete separation of regression models may be mitigated by deliberate model misspecification by merging covariate categories, dropping higher‐order terms, omitting covariates that strongly predict exposure, and so froth. These tradeoffs between different sources of bias will be a topic for future studies.

The present study aimed to highlight the bias introduced into regression standardization by the easy‐to‐use Firth's method, we focused only on the limited approach for addressing (quasi‐)complete separation. For rare event analysis, a wide range of methods exists to handle “sparse‐data bias,” including Bayesian methods such as log‐F(1,1) prior [34] or the Cauchy prior [35], as well as penalized likelihood methods, including lasso or ridge. Moreover, “iterative” adjustment procedures for model fitting can enhance convergence stability [36]. In principle, these methods can be directly incorporated into regression standardization procedures. Evaluating potential bias and its relative performance in rare‐event settings is beyond the scope of this study.

Finally, a wide variety of models can be applied using Firth's method. For parametric models other than logistic models, an iterative algorithm can be used by adjusting the score functions [37]. In practice, we often choose alternative parametric models such as log‐linear Poisson or linear normal models, accompanied by robust sandwich variance estimators. With iterative fitting with Firth's correction of these alternative models, it is unknown whether the problems highlighted in this paper occur in rare event data and modifications such as FLIC/FLAC. Multinomial logit models, which utilize the framework of Poisson log‐linear models, have the potential to expand Firth's method beyond logistic regression [38].

## Author Contributions

Conceptualization and methodology: Tomohiro Shinozaki; Code implementation: Hashibe S; Validation: Wataru Hongo; Writing – original draft: Sotaro Hashibe; Writing – review and editing: Tomohiro Shinozaki; Supervision:Tomohiro Shinozaki. All authors have read and approved the final manuscript.

## Funding

This work was supported by the Japan Society for the Promotion of Science (Grant No. 24K14864) and the Japan Agency for Medical Research and Development (Grant No. JP25mk0121297).

## Disclosure

The authors have nothing to report.

## Conflicts of Interest

The authors declare no conflicts of interest.

## Supporting information




**Data S1.** Supporting Information.

## Data Availability

Data sharing not applicable to this article as no datasets were generated or analysed during the current study.
