# Supplementary material for: Caveats on Using Firth's Penalization in the Model‐Based Regression Standardization for Rare Diseases
Source: Stat Med. 2026 Jun 23;45(15-17):e70644. doi: 10.1002/sim.70644 (PMC13288324; doi:10.1002/sim.70644)
Supplement: Supplementary file 1 — Data S1. Supporting Information. [file SIM-45-0-s001.pdf]

Statistics in Medicine

Supplementary materials for "Caveats on using Firth's penalization in the model-based regression standardization for rare diseases" by Hashibe, Hongo, and Shinozaki

1. Details of the simulation settings

The exposure Z and outcome Y were generated by the Bernoulli distribution, with probabilities defined by Equations 8 and 9 in the main text. The values of the parameters are listed in Supplementary Table 1. Different values were chosen for the intercepts (α_0 and β_0) to achieve the target exposure rate (0.1) and event rate (0.005, 0.05, and 0.2). Supplementary Table 2 provides a summary of each simulation scenario, including exposure rates and event rates, ρ for correlations between covariates, the chosen intercepts (α_0 and β_0), the proportion of datasets where quasi-complete separation occurs (among 1000 datasets), and the proportion of datasets where 0 cell count occurs in Y–Z cross-classification.

Supplementary Table 1. Parameters of the data-generating logistic model (Equations 8 and 9 in the main text)

j	0	1	2	3	4	5	6	7	8	9	10
α_j	α_0	1	1	1	1	1	1	1	1	1	1
β_j	β_0	1	1	1	1	1	1	1	1	1	1

Copyright © **2026** John Wiley & Sons, Ltd.

Supplementary Table 2. Summary of simulation scenarios, number of separations, and zero counts for at least one Y–Z combination in the simulated datasets

Scenario	N	Event rate	Exposure rate	Correlation	β_0	α_0	0 count in (Y, Z)	Number of separations	Separation rate
1	2500	0.005	0.1	0.5	-12.9	-8.9	21	883	0.902
2	2500	0.01	0.1	0.5	-12.2	-8.9	1	469	0.469
3	2500	0.03	0.1	0.5	-10.85	-8.9	0	0	0.000
4	2500	0.05	0.1	0.5	-10.25	-8.9	0	0	0.000
5	2500	0.1	0.1	0.5	-9.1	-8.9	0	0	0.000
6	2500	0.2	0.1	0.5	-7.6	-8.9	0	0	0.000
7	2500	0.005	0.1	0.25	-12.7	-8.4	60	782	0.832
8	2500	0.01	0.1	0.25	-11.5	-8.4	0	69	0.069
9	2500	0.03	0.1	0.25	-10.5	-8.4	0	0	0.000
10	2500	0.05	0.1	0.25	-9.6	-8.4	0	0	0.000
11	2500	0.1	0.1	0.25	-8.5	-8.4	0	0	0.000
12	2500	0.2	0.1	0.25	-7.3	-8.4	0	0	0.000
13	2500	0.005	0.1	0	-11.75	-8	0	240	0.240
14	2500	0.01	0.1	0	-11.2	-8	0	25	0.025
15	2500	0.03	0.1	0	-10.05	-8	0	0	0.000
16	2500	0.05	0.1	0	-9.3	-8	0	0	0.000
17	2500	0.1	0.1	0	-8.25	-8	0	0	0.000
18	2500	0.2	0.1	0	-7.1	-8	0	0	0.000
19	500	0.005	0.1	0.5	-12.24	-8.55	294	702	0.994
20	500	0.01	0.1	0.5	-11.6	-8.55	91	864	0.950
21	500	0.03	0.1	0.5	-10.9	-8.55	12	725	0.734
22	500	0.05	0.1	0.5	-10	-8.55	0	140	0.140
23	500	0.1	0.1	0.5	-8.9	-8.55	0	0	0.000
24	500	0.2	0.1	0.5	-7.55	-8.55	0	0	0.000
25	500	0.005	0.1	0.25	-11.96	-8.2	410	582	0.986
26	500	0.01	0.1	0.25	-11.5	-8.2	193	760	0.942
27	500	0.03	0.1	0.25	-10.3	-8.2	5	361	0.363
28	500	0.05	0.1	0.25	-9.7	-8.2	0	66	0.066
29	500	0.1	0.1	0.25	-8.7	-8.2	0	0	0.000
30	500	0.2	0.1	0.25	-7.2	-8.2	0	0	0.000
31	500	0.005	0.1	0	-11.8	-8.2	493	492	0.970
32	500	0.01	0.1	0	-11.3	-8.2	239	709	0.932
33	500	0.03	0.1	0	-10.05	-8.2	5	282	0.283
34	500	0.05	0.1	0	-9.35	-8.2	0	28	0.028
35	500	0.1	0.1	0	-8.3	-8.2	0	0	0.000
36	500	0.2	0.1	0	-7.03	-8.2	0	0	0.000

Note. Separation rate was calculated as Number of separations$/(1000 - m)$, where m denotes the number of replications in which at least one cell in the (Y, Z) contingency table had zero counts.

 Copyright © 2026 John Wiley & Sons, Ltd.

Supplementary Table 3. True values of each scenario for $\mathrm{E}[Y(1)]$, $\mathrm{E}[Y(0)]$, risk difference ($\mathrm{E}[Y(1)] - \mathrm{E}[Y(0)]$), and log risk ratio ($\log(\mathrm{E}[Y(1)]/\mathrm{E}[Y(0)])$). Using n = 10,000,000, scenarios 19–36 have the same true values as scenarios 1–18.

Scenario	Event rate	Correlation	$\mathrm{E}[\mu(1)]$	$\mathrm{E}[\mu(0)]$	$\mathrm{E}[\mu(1)] - \mathrm{E}[\mu(0)]$	$\log(\mathrm{E}[\mu(1)]) - \log(\mathrm{E}[\mu(0)])$
1	0.005	0.5	0.007	0.003	0.004	0.772
2	0.010	0.5	0.013	0.006	0.007	0.748
3	0.030	0.5	0.040	0.020	0.019	0.661
4	0.050	0.5	0.062	0.034	0.028	0.607
5	0.100	0.5	0.129	0.079	0.051	0.496
6	0.200	0.5	0.271	0.188	0.083	0.365
7	0.005	0.25	0.005	0.002	0.003	0.780
8	0.010	0.25	0.014	0.007	0.008	0.745
9	0.030	0.25	0.034	0.017	0.017	0.690
10	0.050	0.25	0.070	0.037	0.032	0.622
11	0.100	0.25	0.146	0.087	0.060	0.524
12	0.200	0.25	0.279	0.185	0.094	0.413
13	0.005	0	0.008	0.004	0.004	0.772
14	0.010	0	0.014	0.006	0.007	0.757
15	0.030	0	0.038	0.019	0.019	0.703
16	0.050	0	0.070	0.037	0.034	0.651
17	0.100	0	0.149	0.085	0.064	0.559
18	0.200	0	0.289	0.185	0.104	0.444

Copyright © **2026** John Wiley & Sons, Ltd.

2. Additional simulation results

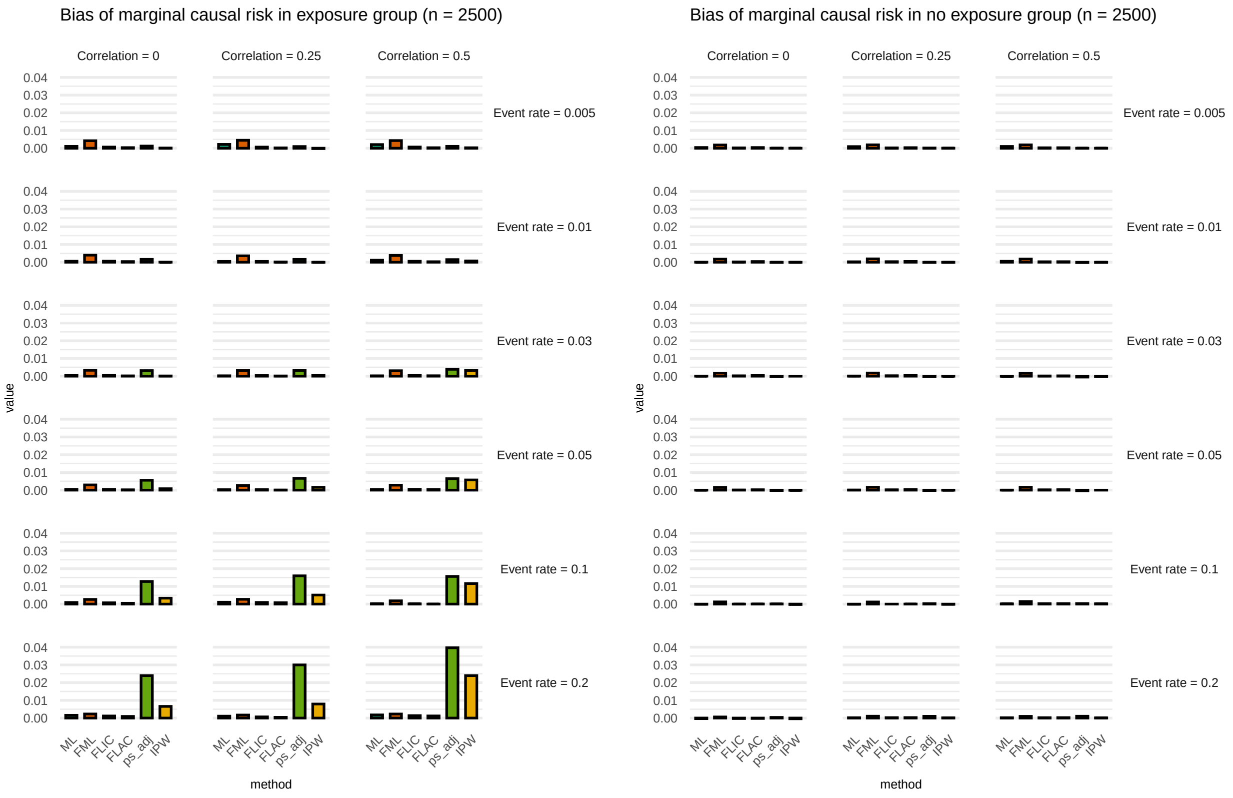

Supplementary Figure 1–1. Bar plots of bias for $\mathrm{E}[Y(1)]$ and $\mathrm{E}[Y(0)]$ (N = 2500). The compared methods are regression standardization with maximum likelihood estimates (ML), ML not adjusted for confounding variables (Unadj), regression standardization following Firth's method (FML), regression standardization following Firth's method with corresponding modification (FLIC, FLAC), regression standardization with propensity score–adjusted model (PS-adj), and inverse probability weighted estimates (IPW).

Copyright © **2026** John Wiley & Sons, Ltd.

Prepared using simauth.cls

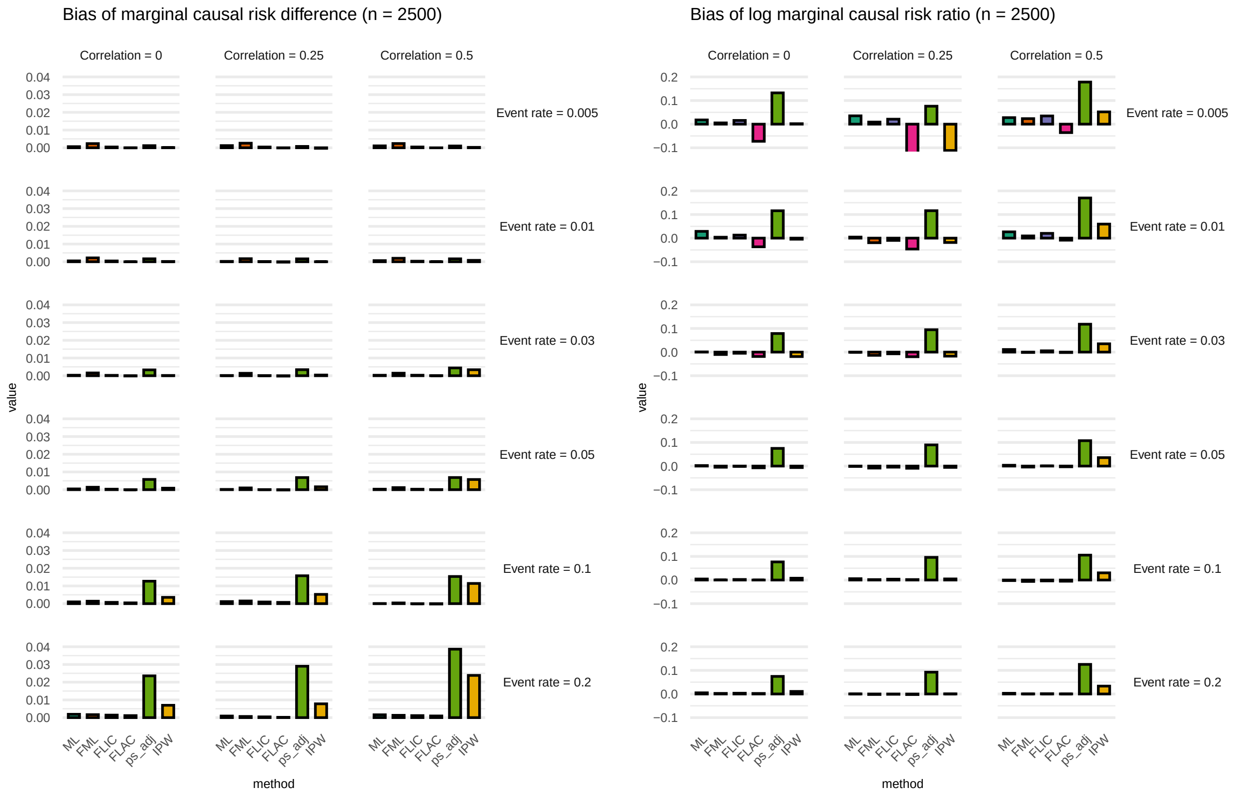

Supplementary Figure 1–2. Bar plots of bias for $\mathrm{E}[Y(1)] - \mathrm{E}[Y(0)]$ and $\log(\mathrm{E}[Y(1)]) - \log(\mathrm{E}[Y(0)])$ (N = 2500). The compared methods are regression standardization with maximum likelihood estimates (ML), ML not adjusted for confounding variables (Unadj), regression standardization following Firth's method (FML), regression standardization following Firth's method with corresponding modification (FLIC, FLAC), regression standardization with propensity score–adjusted model (PS-adj), and inverse probability weighted estimates (IPW).

Copyright © **2026** John Wiley & Sons, Ltd.

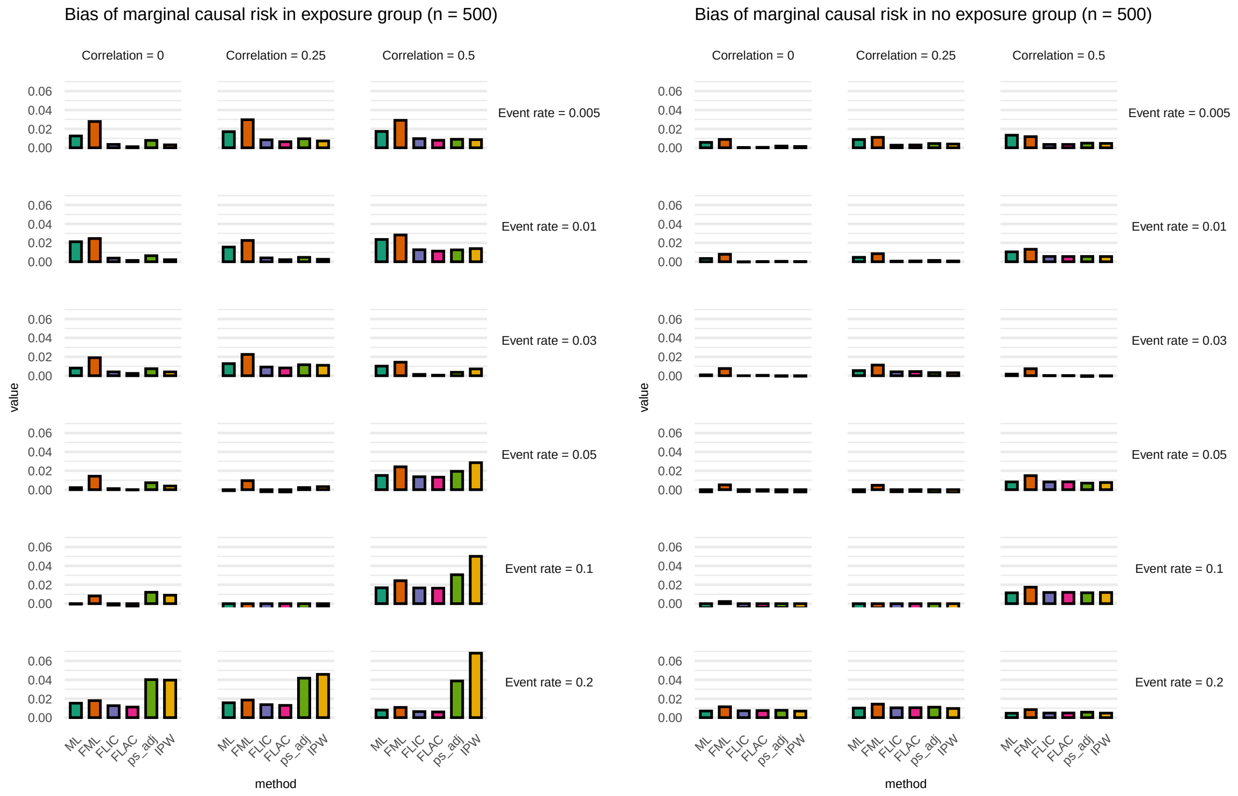

Supplementary Figure 1–3. Bar plots of bias for $E[Y(1)]$ and $E[Y(0)]$ (N = 500). The compared methods are regression standardization with maximum likelihood estimates (ML), ML not adjusted for confounding variables (Unadj), regression standardization following Firth's method (FML), regression standardization following Firth's method with corresponding modification (FLIC, FLAC), regression standardization with propensity score–adjusted model (PS-adj), and inverse probability weighted estimates (IPW).

 Copyright © 2026 John Wiley & Sons, Ltd.

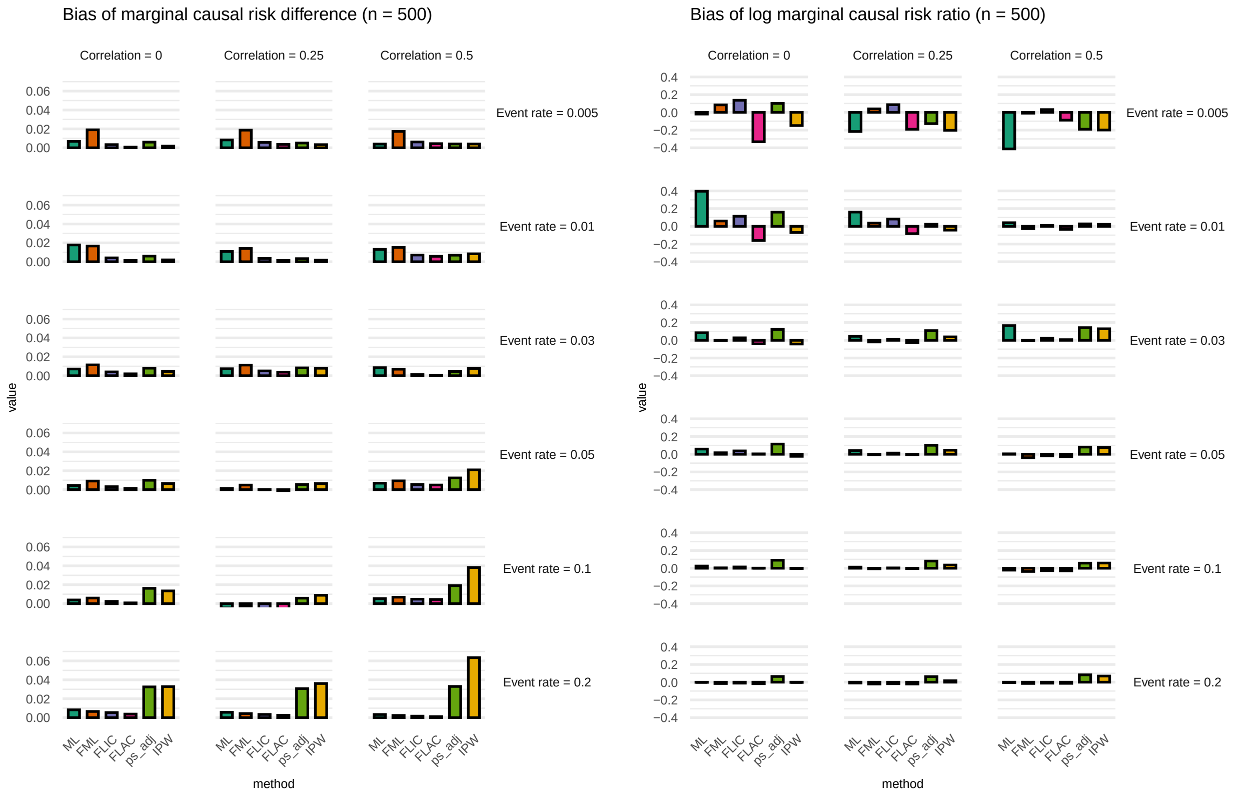

Supplementary Figure 1–4. Bar plots of bias for $\mathrm{E}[Y(1)] - \mathrm{E}[Y(0)]$ and $\log(\mathrm{E}[Y(1)]) - \log(\mathrm{E}[Y(0)])$ (N = 500). The compared methods are regression standardization with maximum likelihood estimates (ML), ML not adjusted for confounding variables (Unadj), regression standardization following Firth's method (FML), regression standardization following Firth's method with corresponding modification (FLIC, FLAC), regression standardization with propensity score–adjusted model (PS-adj), and inverse probability weighted estimates (IPW).

Copyright © **2026** John Wiley & Sons, Ltd.

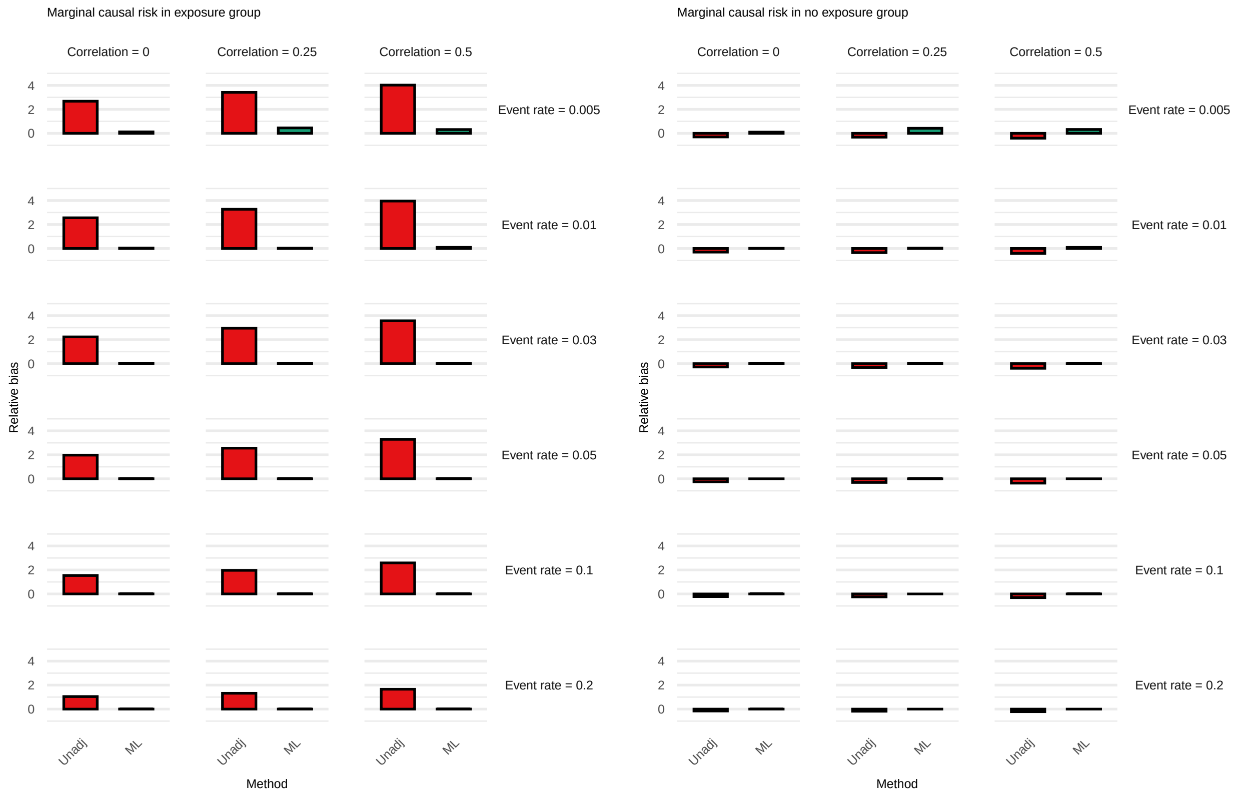

Supplementary Figure 2–1. Relative bias of $\mathrm{E}[Y(1)]$ and $\mathrm{E}[Y(0)]$ (N = 2500). The compared methods are regression standardization with maximum likelihood estimates (ML) and ML not adjusted for confounding variables (Unadj).

Copyright © 2026 John Wiley & Sons, Ltd.

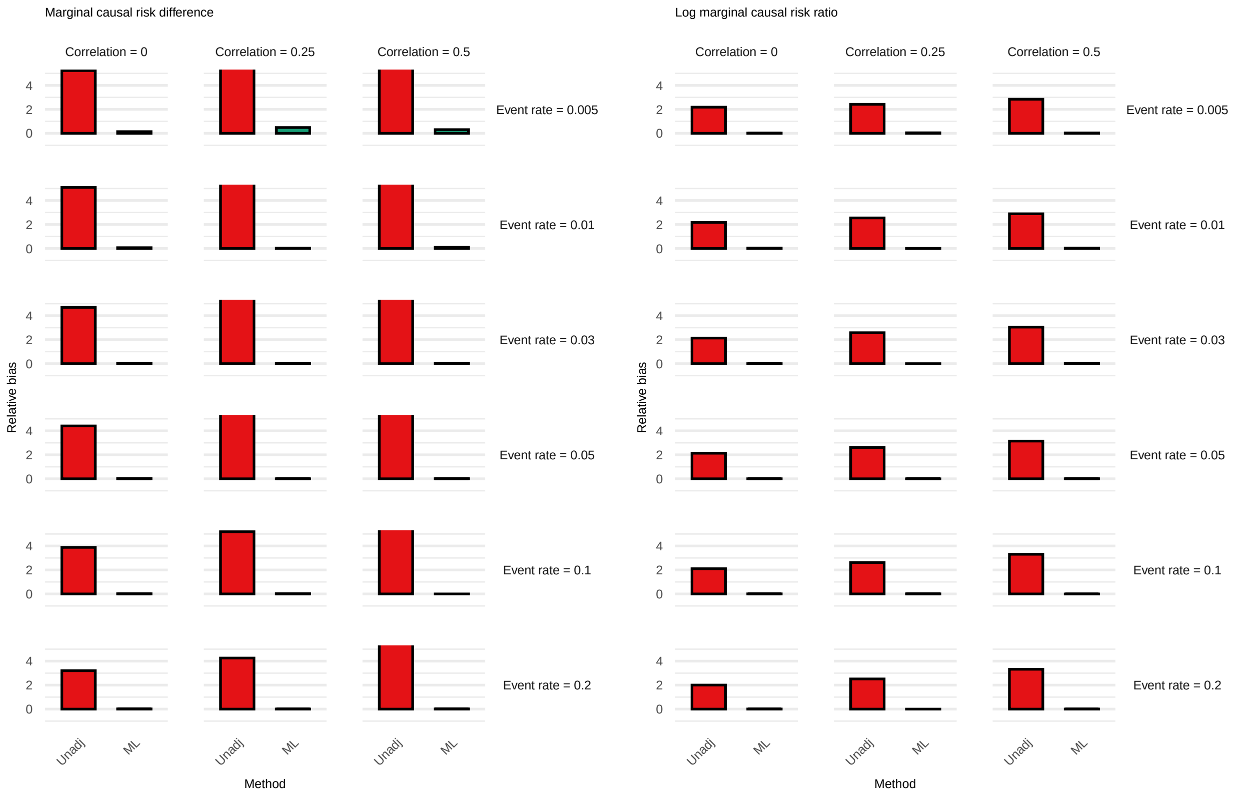

Supplementary Figure 2–2. Relative bias of $\mathrm{E}[Y(1)] - \mathrm{E}[Y(0)]$ and $\log(\mathrm{E}[Y(1)]) - \log(\mathrm{E}[Y(0)])$ (N = 2500). The compared methods are regression standardization with maximum likelihood estimates (ML) and ML not adjusted for confounding variables (Unadj).

Copyright © **2026** John Wiley & Sons, Ltd.

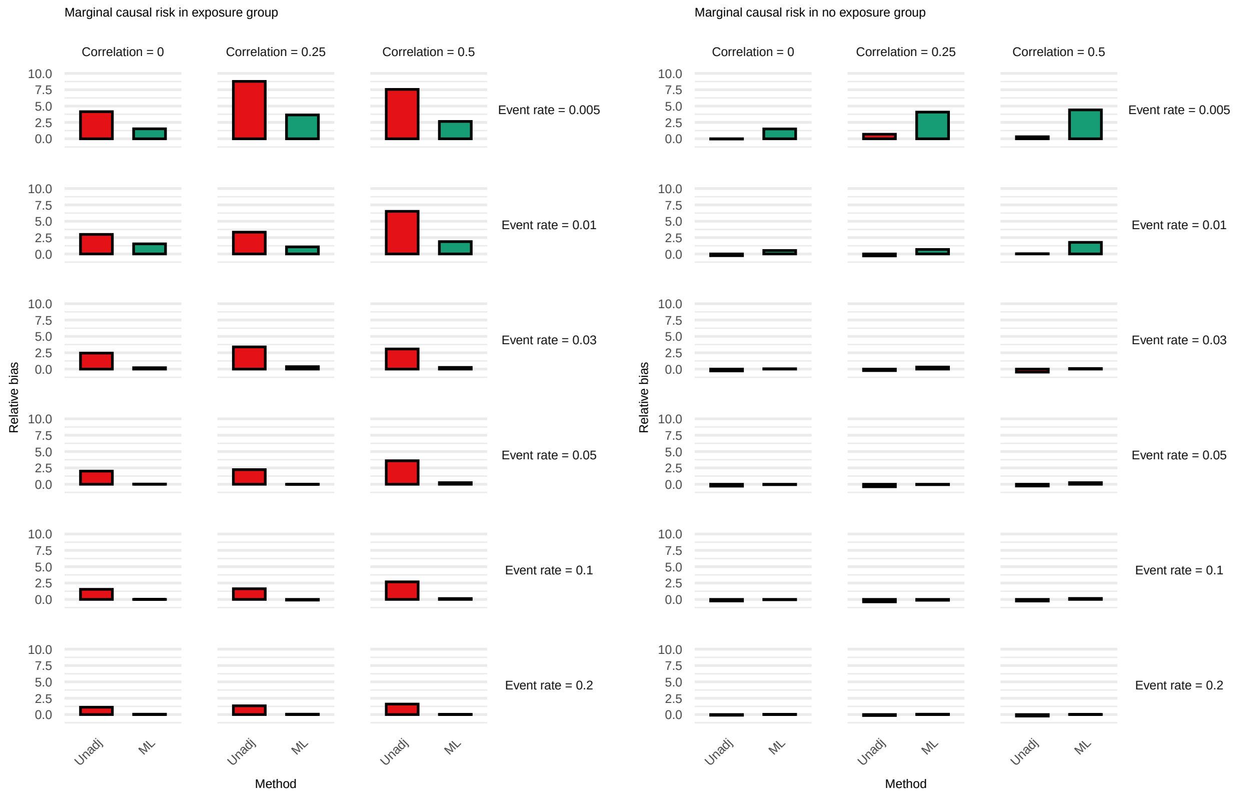

Supplementary Figure 2–3. Relative bias of $\mathrm{E}[Y(1)]$ and $\mathrm{E}[Y(0)]$ (N = 500). The compared methods are regression standardization with maximum likelihood estimates (ML) and ML not adjusted for confounding variables (Unadj).

Copyright © 2026 John Wiley & Sons, Ltd.

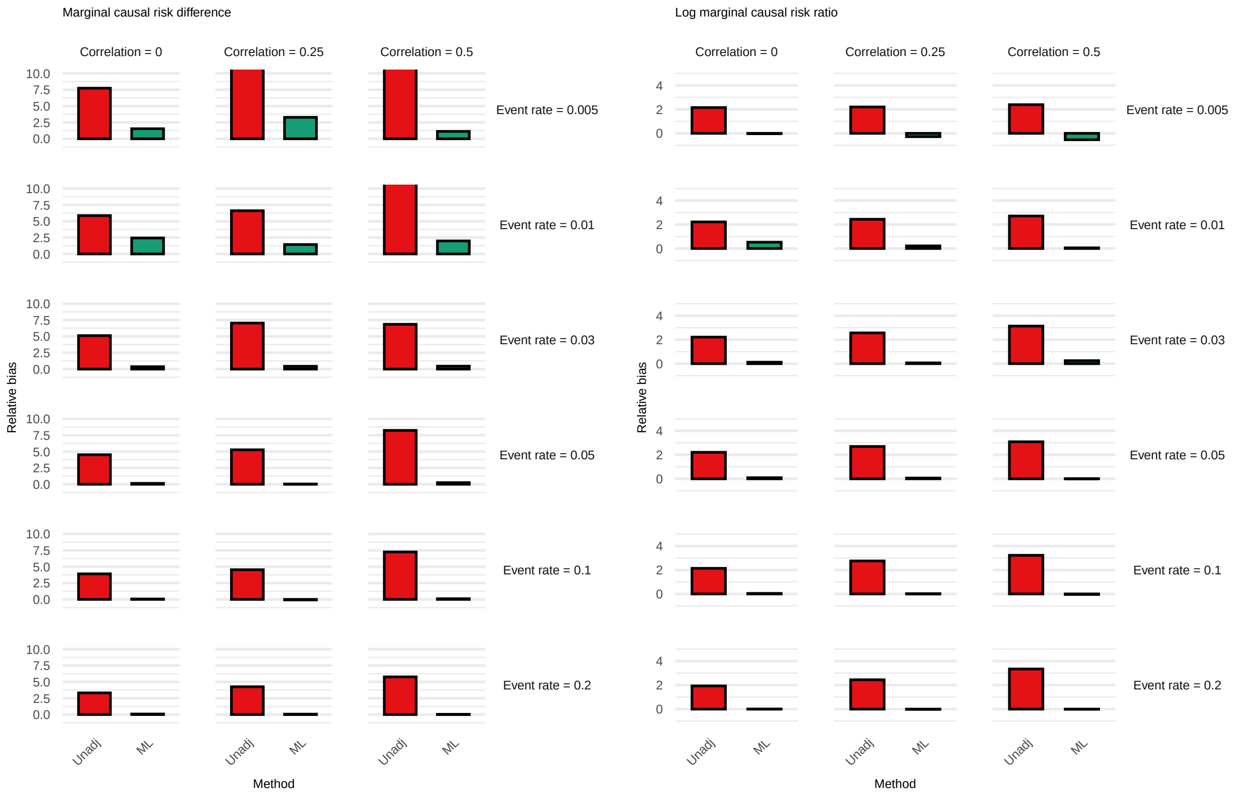

Supplementary Figure 2–4. Relative bias of $\mathrm{E}[Y(1)] - \mathrm{E}[Y(0)]$ and $\log(\mathrm{E}[Y(1)]) - \log(\mathrm{E}[Y(0)])$ (N = 500). The compared methods are regression standardization with maximum likelihood estimates (ML) and ML not adjusted for confounding variables (Unadj).

Copyright © 2026 John Wiley & Sons, Ltd.

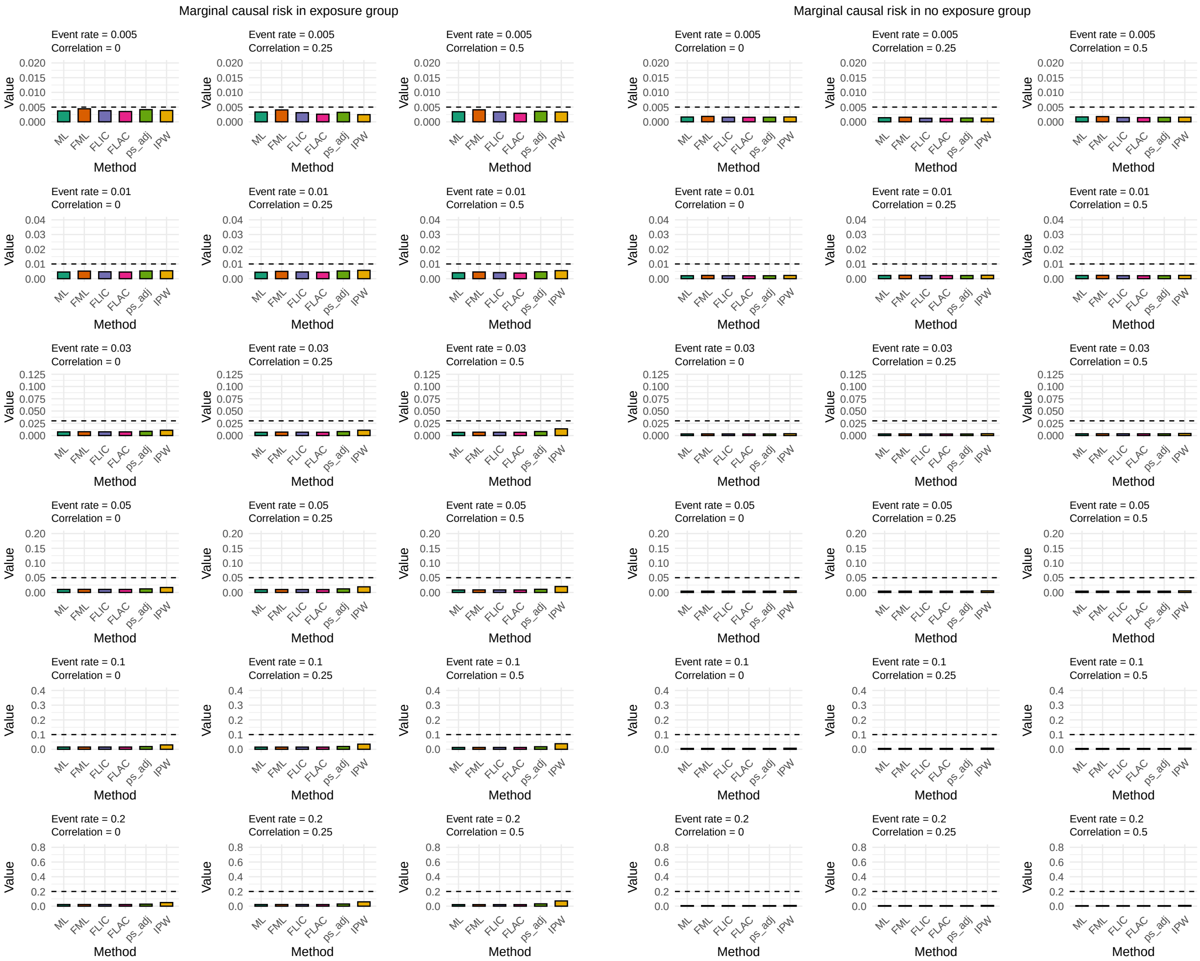

Supplementary Figure 3–1. Bar plots of MESE for $\mathrm{E}[Y(1)]$ and $\mathrm{E}[Y(0)]$ (N = 2500). The compared methods are regression standardization with maximum likelihood estimates (ML), ML not adjusted for confounding variables (Unadj), regression standardization following Firth's method (FML), regression standardization following Firth's method with corresponding modification (FLIC, FLAC), regression standardization with propensity score–adjusted model (PS-adj), and inverse probability weighted estimates (IPW).

Copyright © 2026 John Wiley & Sons, Ltd.

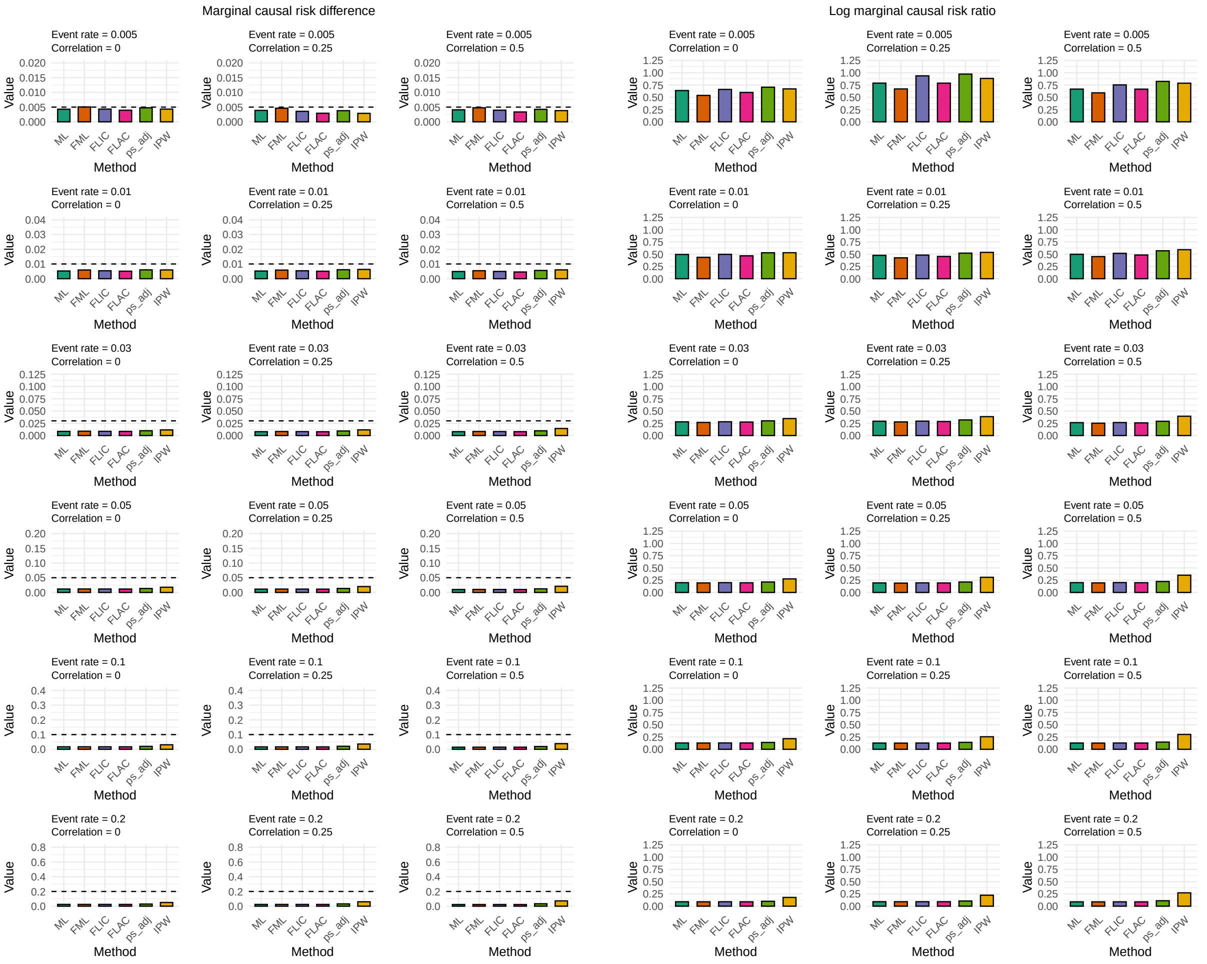

Supplementary Figure 3–2. Bar plots of MESE for $\mathrm{E}[Y(1)] - \mathrm{E}[Y(0)]$ and $\log(\mathrm{E}[Y(1)]) - \log(\mathrm{E}[Y(0)])$ (N = 2500). The compared methods are regression standardization with maximum likelihood estimates (ML), ML not adjusted for confounding variables (Unadj), regression standardization following Firth's method (FML), regression standardization following Firth's method with corresponding modification (FLIC, FLAC), regression standardization with propensity score–adjusted model (PS-adj), and inverse probability weighted estimates (IPW).

Copyright © **2026** John Wiley & Sons, Ltd.

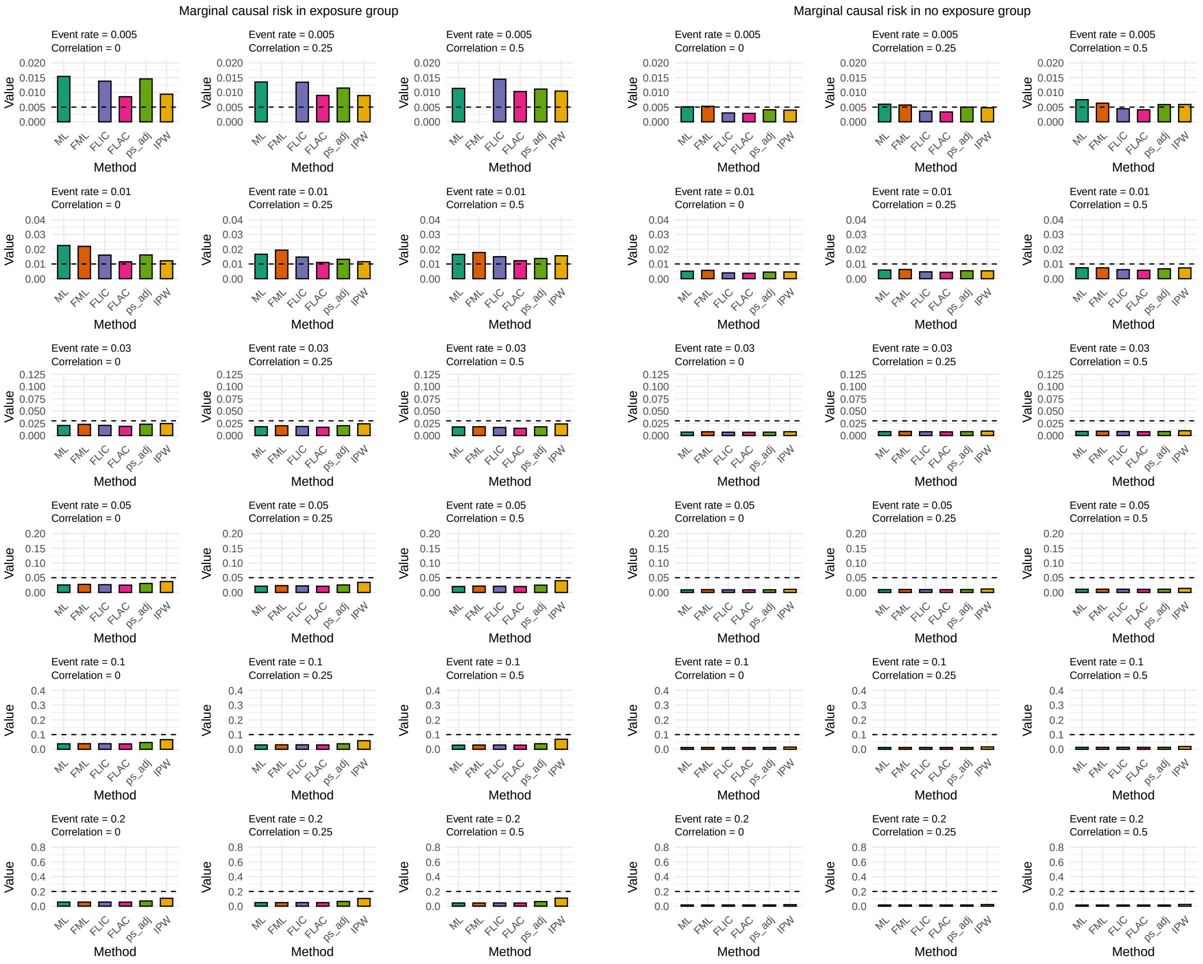

Supplementary Figure 3–3. Bar plots of MESE for $\mathrm{E}[Y(1)]$ and $\mathrm{E}[Y(0)]$ (N = 500). The compared methods are regression standardization with maximum likelihood estimates (ML), ML not adjusted for confounding variables (Unadj), regression standardization following Firth's method (FML), regression standardization following Firth's method with corresponding modification (FLIC, FLAC), regression standardization with propensity score–adjusted model (PS-adj), and inverse probability weighted estimates (IPW). Values exceeding the y-axis maximum are not displayed.

Copyright © 2026 John Wiley & Sons, Ltd.

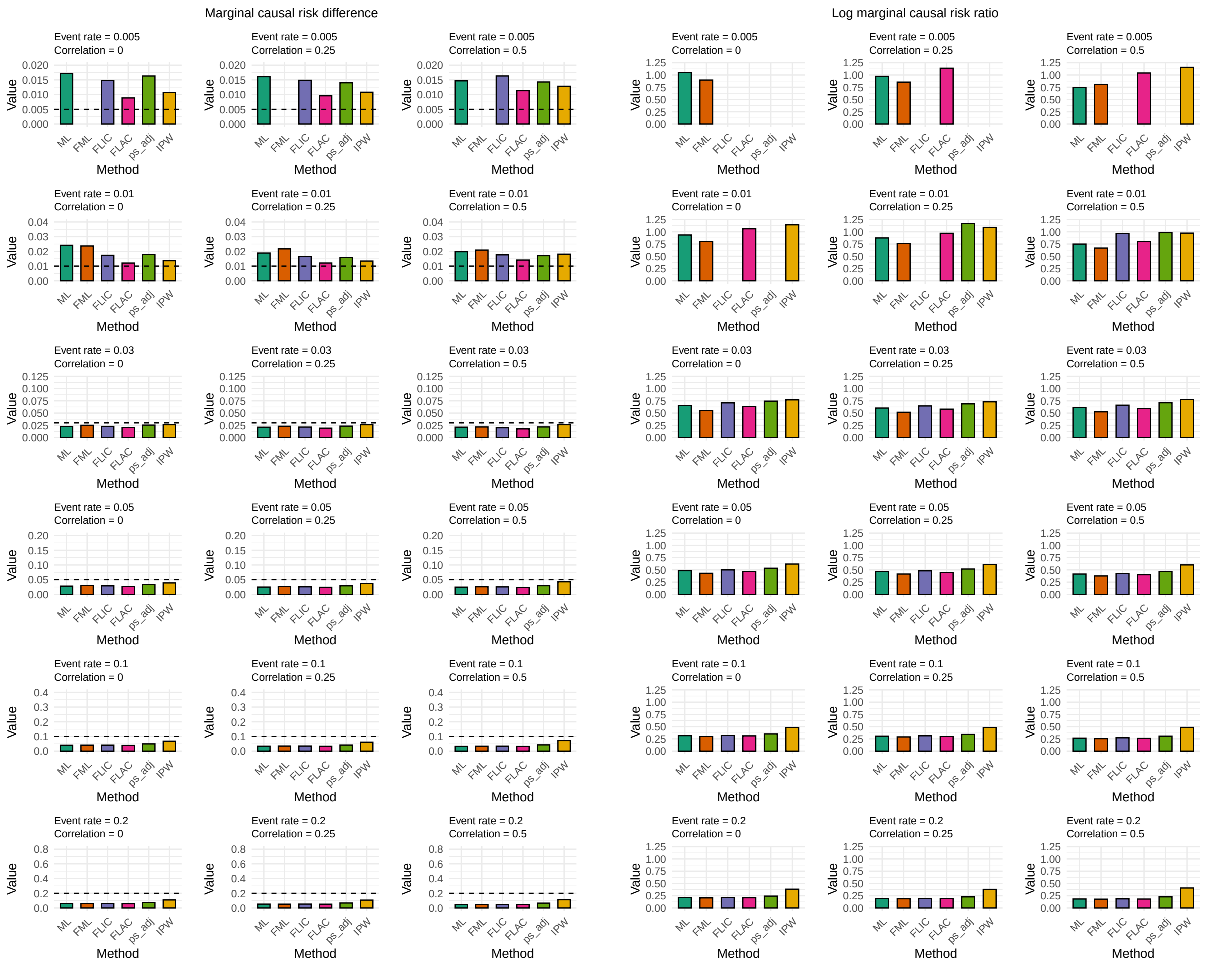

Supplementary Figure 3–4. Bar plots of MESE for $\mathrm{E}[Y(1)] - \mathrm{E}[Y(0)]$ and $\log(\mathrm{E}[Y(1)]) - \log(\mathrm{E}[Y(0)])$ (N = 500). The compared methods are regression standardization with maximum likelihood estimates (ML), ML not adjusted for confounding variables (Unadj), regression standardization following Firth's method (FML), regression standardization following Firth's method with corresponding modification (FLIC, FLAC), regression standardization with propensity score–adjusted model (PS-adj), and inverse probability weighted estimates (IPW). Values exceeding the y-axis maximum are not displayed.

Copyright © 2026 John Wiley & Sons, Ltd.

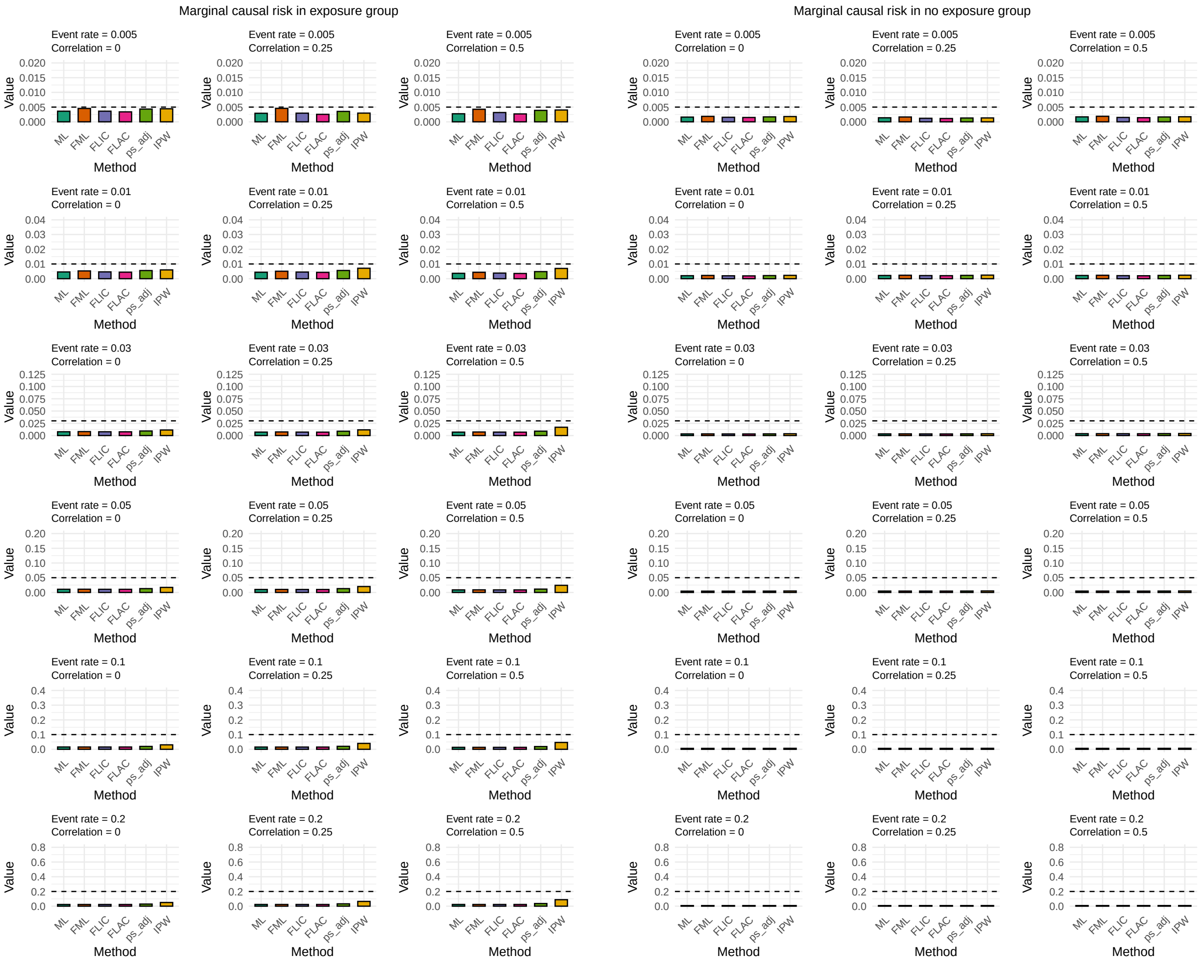

Supplementary Figure 4–1. Bar plots of MCSE for $\mathrm{E}[Y(1)]$ and $\mathrm{E}[Y(0)]$ (N = 2500). The compared methods are regression standardization with maximum likelihood estimates (ML), ML not adjusted for confounding variables (Unadj), regression standardization following Firth's method (FML), regression standardization following Firth's method with corresponding modification (FLIC, FLAC), regression standardization with propensity score–adjusted model (PS-adj), and inverse probability weighted estimates (IPW).

Copyright © **2026** John Wiley & Sons, Ltd.

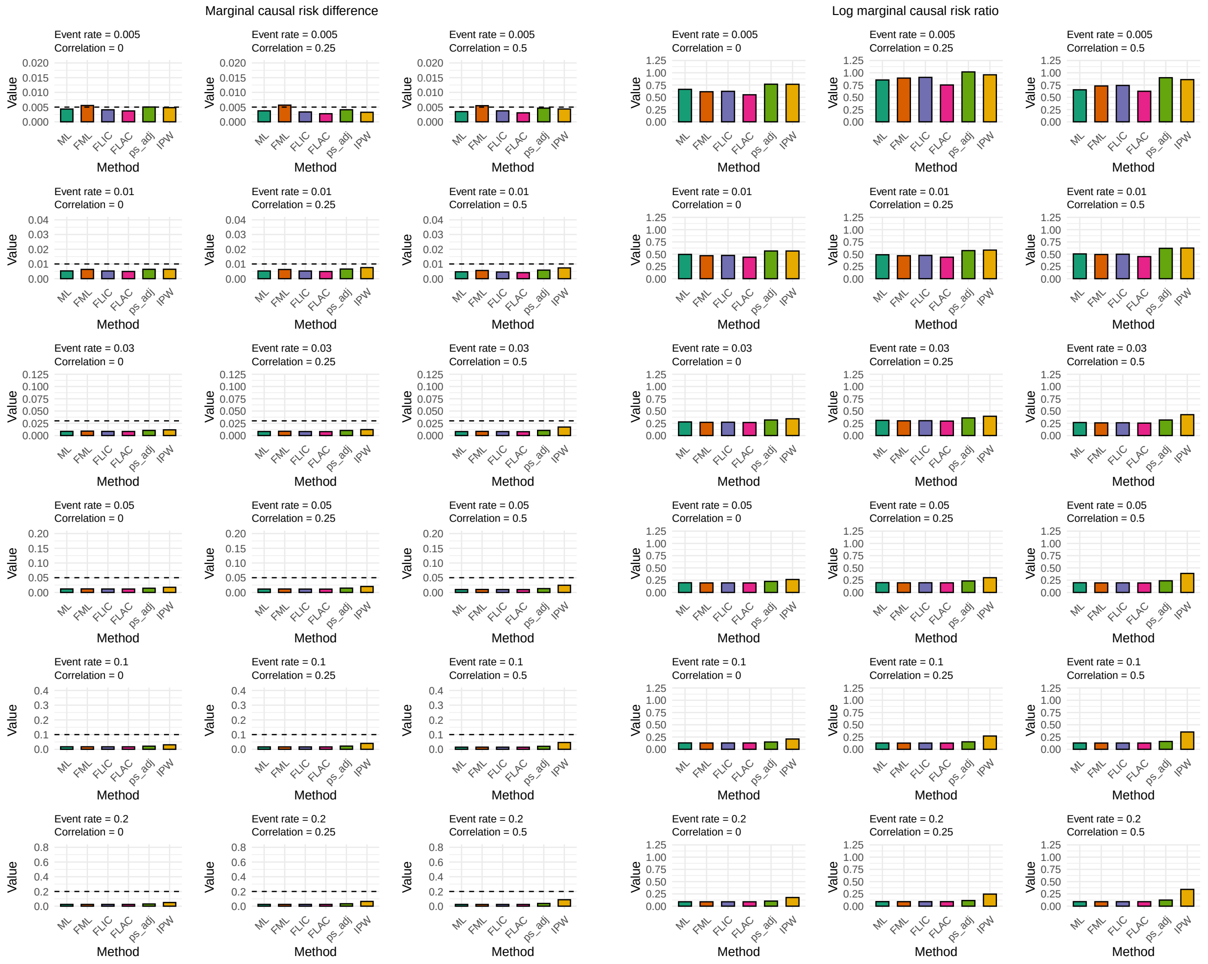

Supplementary Figure 4–2. Bar plots of MCSE for $\mathrm{E}[Y(1)] - \mathrm{E}[Y(0)]$ and $\log(\mathrm{E}[Y(1)]) - \log(\mathrm{E}[Y(0)])$ (N = 2500). The compared methods are regression standardization with maximum likelihood estimates (ML), ML not adjusted for confounding variables (Unadj), regression standardization following Firth's method (FML), regression standardization following Firth's method with corresponding modification (FLIC, FLAC), regression standardization with propensity score–adjusted model (PS-adj), and inverse probability weighted estimates (IPW).

Copyright © 2026 John Wiley & Sons, Ltd.

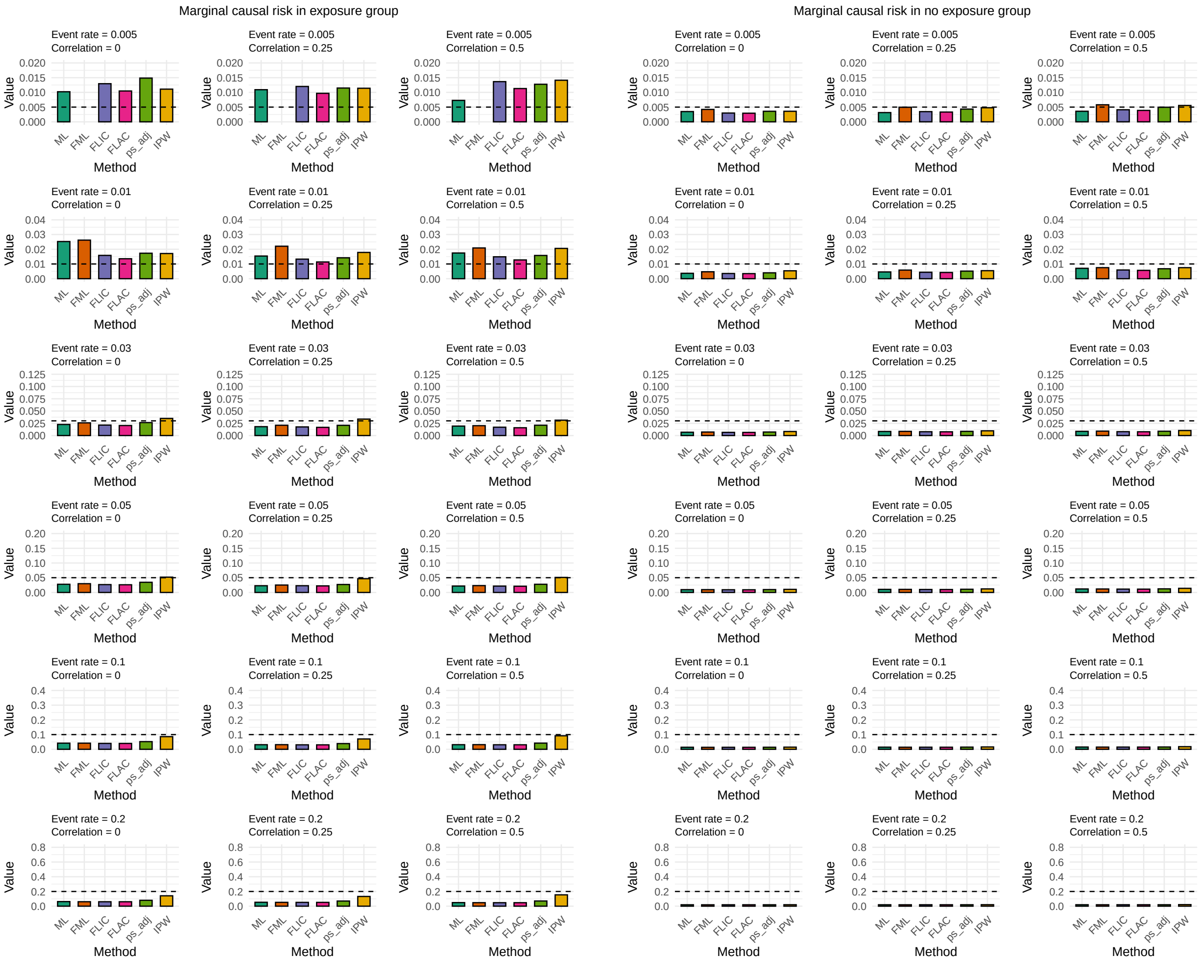

Supplementary Figure 4–3. Bar plots of MCSE for $\mathrm{E}[Y(1)]$ and $\mathrm{E}[Y(0)]$ (N = 500). The compared methods are regression standardization with maximum likelihood estimates (ML), ML not adjusted for confounding variables (Unadj), regression standardization following Firth's method (FML), regression standardization following Firth's method with corresponding modification (FLIC, FLAC), regression standardization with propensity score–adjusted model (PS-adj), and inverse probability weighted estimates (IPW). Values exceeding the y-axis maximum are not displayed.

 Copyright © 2026 John Wiley & Sons, Ltd.

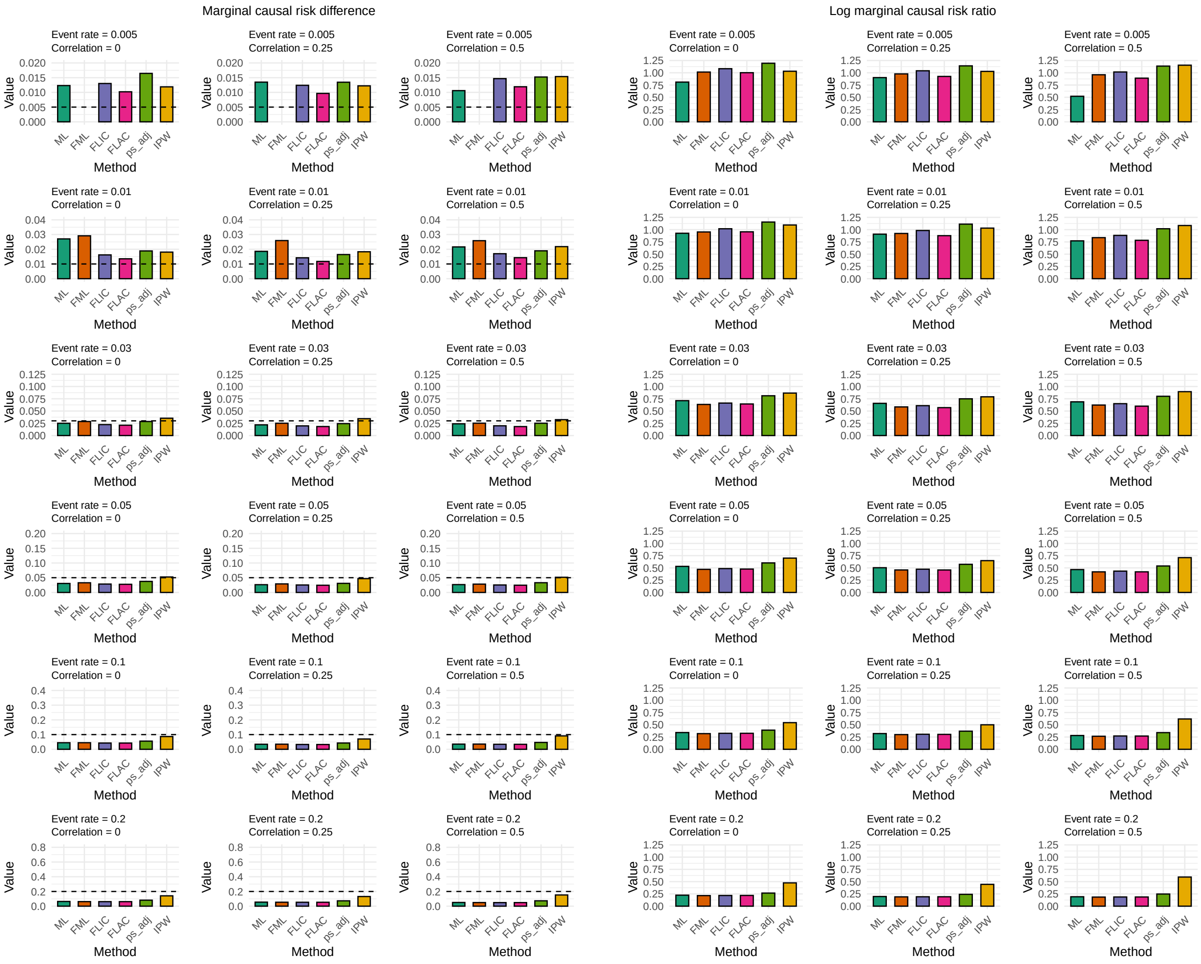

Supplementary Figure 4–4. Bar plots of MCSE for $\mathrm{E}[Y(1)] - \mathrm{E}[Y(0)]$ and $\log(\mathrm{E}[Y(1)]) - \log(\mathrm{E}[Y(0)])$ (N = 500). The compared methods are regression standardization with maximum likelihood estimates (ML), ML not adjusted for confounding variables (Unadj), regression standardization following Firth's method (FML), regression standardization following Firth's method with corresponding modification (FLIC, FLAC), regression standardization with propensity score–adjusted model (PS-adj), and inverse probability weighted estimates (IPW). Values exceeding the y-axis maximum are not displayed.

Copyright © **2026** John Wiley & Sons, Ltd.

Supplementary Table 3–1. Coverage proportion (95% CI) for E$[Y(1)]$ and E$[Y(0)]$ (N = 2500).

Scenario	N	Event rate	Correlation	Coverage proportion (95% CI) for											
				E$[Y(1)]$						E$[Y(0)]$					
				ML	FML	FLIC	FLAC	PS-adj	IPW	ML	FML	FLIC	FLAC	PS-adj	IPW
1	2500	0.005	0.5	0.096	1	1	1	0.979	0.979	0.096	1	1	1	0.979	0.979
2	2500	0.010	0.5	0.530	1	1	1	0.999	0.999	0.530	1	1	1	0.999	0.999
3	2500	0.030	0.5	1	1	1	1	1	1	1	1	1	1	1	1
4	2500	0.050	0.5	1	1	1	1	1	1	1	1	1	1	1	1
5	2500	0.100	0.5	1	1	1	1	1	1	1	1	1	1	1	1
6	2500	0.200	0.5	1	1	1	1	1	1	1	1	1	1	1	1
7	2500	0.005	0.25	0.158	1	1	1	0.940	0.940	0.158	1	1	1	0.940	0.940
8	2500	0.010	0.25	0.931	1	1	1	1	1	0.931	1	1	1	1	1
9	2500	0.030	0.25	1	1	1	1	1	1	1	1	1	1	1	1
10	2500	0.050	0.25	1	1	1	1	1	1	1	1	1	1	1	1
11	2500	0.100	0.25	1	1	1	1	1	1	1	1	1	1	1	1
12	2500	0.200	0.25	1	1	1	1	1	1	1	1	1	1	1	1
13	2500	0.005	0	0.760	1	1	1	1	1	0.760	1	1	1	1	1
14	2500	0.010	0	0.975	1	1	1	1	1	0.975	1	1	1	1	1
15	2500	0.030	0	1	1	1	1	1	1	1	1	1	1	1	1
16	2500	0.050	0	1	1	1	1	1	1	1	1	1	1	1	1
17	2500	0.100	0	1	1	1	1	1	1	1	1	1	1	1	1
18	2500	0.200	0	1	1	1	1	1	1	1	1	1	1	1	1

Copyright © 2026 John Wiley & Sons, Ltd.

Supplementary Table 3–2. Coverage proportion (95% CI) for $\mathrm{E}[Y(1)] - \mathrm{E}[Y(0)]$ and $\log(\mathrm{E}[Y(1)]) - \log(\mathrm{E}[Y(0)])$ (N = 2500).

Scenario	N	Event rate	Correlation	Coverage proportion (95% CI) for											
				$\mathrm{E}[Y(1)] - \mathrm{E}[Y(0)]$						$\log(\mathrm{E}[Y(1)]) - \log(\mathrm{E}[Y(0)])$					
				ML	FML	FLIC	FLAC	PS-adj	IPW	ML	FML	FLIC	FLAC	PS-adj	IPW
1	2500	0.005	0.5	0.096	1	1	1	0.979	0.979	0.096	1	1	1	0.979	0.979
2	2500	0.010	0.5	0.530	1	1	1	0.999	0.999	0.530	1	1	1	0.999	0.999
3	2500	0.030	0.5	1	1	1	1	1	1	1	1	1	1	1	1
4	2500	0.050	0.5	1	1	1	1	1	1	1	1	1	1	1	1
5	2500	0.100	0.5	1	1	1	1	1	1	1	1	1	1	1	1
6	2500	0.200	0.5	1	1	1	1	1	1	1	1	1	1	1	1
7	2500	0.005	0.25	0.158	1	1	1	0.940	0.940	0.158	1	1	1	0.940	0.940
8	2500	0.010	0.25	0.931	1	1	1	1	1	0.931	1	1	1	1	1
9	2500	0.030	0.25	1	1	1	1	1	1	1	1	1	1	1	1
10	2500	0.050	0.25	1	1	1	1	1	1	1	1	1	1	1	1
11	2500	0.100	0.25	1	1	1	1	1	1	1	1	1	1	1	1
12	2500	0.200	0.25	1	1	1	1	1	1	1	1	1	1	1	1
13	2500	0.005	0	0.760	1	1	1	1	1	0.760	1	1	1	1	1
14	2500	0.010	0	0.975	1	1	1	1	1	0.975	1	1	1	1	1
15	2500	0.030	0	1	1	1	1	1	1	1	1	1	1	1	1
16	2500	0.050	0	1	1	1	1	1	1	1	1	1	1	1	1
17	2500	0.100	0	1	1	1	1	1	1	1	1	1	1	1	1
18	2500	0.200	0	1	1	1	1	1	1	1	1	1	1	1	1

Copyright © **2026** John Wiley & Sons, Ltd.

Supplementary Table 3–3. Coverage proportion (95% CI) for $\mathrm{E}[Y(1)]$ and $\mathrm{E}[Y(0)]$ (N = 500).

Scenario	N	Event rate	Correlation	Coverage proportion (95% CI) for											
				$\mathrm{E}[Y(1)]$						$\mathrm{E}[Y(0)]$					
				ML	FML	FLIC	FLAC	PS-adj	IPW	ML	FML	FLIC	FLAC	PS-adj	IPW
19	500	0.005	0.5	0.004	1	1	1	0.706	0.706	0.004	1	1	1	0.706	0.706
20	500	0.010	0.5	0.045	1	1	1	0.909	0.909	0.045	1	1	1	0.909	0.909
21	500	0.030	0.5	0.263	1	1	1	0.988	NA	0.263	1	1	1	0.988	0.988
22	500	0.050	0.5	0.860	1	1	1	1	1	0.860	1	1	1	1	1
23	500	0.100	0.5	1	1	1	1	1	1	1	1	1	1	1	1
24	500	0.200	0.5	1	1	1	1	1	NA	1	1	1	1	1	1
25	500	0.005	0.25	0.008	1	1	1	0.590	0.590	0.008	1	1	1	0.590	0.590
26	500	0.010	0.25	0.047	1	1	1	0.807	0.807	0.047	1	1	1	0.807	0.807
27	500	0.030	0.25	0.634	1	1	1	0.995	0.995	0.634	1	1	1	0.995	0.995
28	500	0.050	0.25	0.934	1	1	1	1	1	0.934	1	1	1	1	1
29	500	0.100	0.25	1	1	1	1	1	1	1	1	1	1	1	1
30	500	0.200	0.25	1	1	1	1	1	1	1	1	1	1	1	1
31	500	0.005	0	0.015	1	1	1	0.507	0.507	0.015	1	1	1	0.507	0.507
32	500	0.010	0	0.052	1	1	1	0.761	0.761	0.052	1	1	1	0.761	0.761
33	500	0.030	0	0.713	1	1	1	0.995	0.994	0.713	1	1	1	0.995	0.995
34	500	0.050	0	0.972	1	1	1	1	NA	0.972	1	1	1	1	1
35	500	0.100	0	1	1	1	1	1	1	1	1	1	1	1	1
36	500	0.200	0	1	1	1	1	1	NA	1	1	1	1	1	1

 Copyright © 2026 John Wiley & Sons, Ltd.

Supplementary Table 3–4. Coverage proportion (95% CI) for $\mathrm{E}[Y(1)] - \mathrm{E}[Y(0)]$ and $\log(\mathrm{E}[Y(1)]) - \log(\mathrm{E}[Y(0)])$ (N = 500).

Scenario	N	Event rate	Correlation	Coverage proportion (95% CI) for											
				$\mathrm{E}[Y(1)] - \mathrm{E}[Y(0)]$						$\log(\mathrm{E}[Y(1)]) - \log(\mathrm{E}[Y(0)])$					
				ML	FML	FLIC	FLAC	PS-adj	IPW	ML	FML	FLIC	FLAC	PS-adj	IPW
19	500	0.005	0.5	0.004	1	1	1	0.706	0.706	0.004	1	1	1	0.706	0.706
20	500	0.010	0.5	0.045	1	1	1	0.909	0.909	0.045	1	1	1	0.909	0.909
21	500	0.030	0.5	0.263	1	1	1	0.988	NA	0.263	1	1	1	0.988	NA
22	500	0.050	0.5	0.860	1	1	1	1	1	0.860	1	1	1	1	1
23	500	0.100	0.5	1	1	1	1	1	1	1	1	1	1	1	1
24	500	0.200	0.5	1	1	1	1	1	NA	1	1	1	1	1	NA
25	500	0.005	0.25	0.008	1	1	1	0.590	0.590	0.008	1	1	1	0.590	0.590
26	500	0.010	0.25	0.047	1	1	1	0.807	0.807	0.047	1	1	1	0.807	0.807
27	500	0.030	0.25	0.634	1	1	1	0.995	0.995	0.634	1	1	1	0.995	0.995
28	500	0.050	0.25	0.934	1	1	1	1	1	0.934	1	1	1	1	1
29	500	0.100	0.25	1	1	1	1	1	1	1	1	1	1	1	1
30	500	0.200	0.25	1	1	1	1	1	1	1	1	1	1	1	1
31	500	0.005	0	0.015	1	1	1	0.507	0.507	0.015	1	1	1	0.507	0.507
32	500	0.010	0	0.052	1	1	1	0.761	0.761	0.052	1	1	1	0.761	0.761
33	500	0.030	0	0.713	1	1	1	0.995	0.995	0.713	1	1	1	0.995	0.995
34	500	0.050	0	0.972	1	1	1	1	NA	0.972	1	1	1	1	NA
35	500	0.100	0	1	1	1	1	1	1	1	1	1	1	1	1
36	500	0.200	0	1	1	1	1	1	NA	1	1	1	1	1	NA

Copyright © 2026 John Wiley & Sons, Ltd.

Supplementary Table 4–1. Ratio of MCSE to MESE for $\mathrm{E}[Y(1)]$ and $\mathrm{E}[Y(0)]$.

Scenario	N	Ratio of MCSE to MESE for													
		$\mathrm{E}[Y(1)]$							$\mathrm{E}[Y(0)]$						
		ML	Unadj	FML	FLIC	FLAC	PS-adj	IPW	ML	Unadj	FML	FLIC	FLAC	PS-adj	IPW
1	2500	0.799	1.012	1.006	1.005	1.043	1.059	0.932	1.007	0.927	0.984	1.089	1.090	1.201	1.094
2	2500	0.912	1.005	0.974	1.006	0.955	1.022	0.922	0.995	0.920	0.984	1.024	1.082	1.276	1.059
3	2500	1.058	1.070	1.029	0.988	1.044	1.054	1.035	1.050	1.039	1.050	1.086	1.113	1.241	1.001
4	2500	1.035	1.051	0.994	0.952	1.026	1.039	1.018	1.038	1.022	1.039	1.060	1.097	1.201	0.935
5	2500	1.051	1.158	0.990	1.009	1.046	1.149	1.040	1.150	1.045	1.151	1.074	1.212	1.236	0.949
6	2500	1.097	1.259	1.038	1.002	1.094	1.255	1.089	1.255	1.094	1.255	1.135	1.287	1.237	0.905
7	2500	0.870	0.983	1.041	0.956	1.128	1.052	0.947	0.988	0.979	0.965	1.098	1.010	1.212	1.045
8	2500	1.003	0.995	1.006	0.994	1.019	1.002	0.985	0.978	0.983	0.965	1.063	1.048	1.251	1.032
9	2500	1.046	1.041	1.038	0.988	1.041	1.026	1.025	1.020	1.027	1.015	1.086	1.078	1.069	0.989
10	2500	1.031	1.062	1.004	0.999	1.026	1.051	1.016	1.050	1.021	1.051	1.074	1.098	1.049	0.950
11	2500	1.020	1.101	0.970	0.985	1.016	1.093	1.010	1.095	1.015	1.096	1.059	1.137	1.145	0.913
12	2500	1.040	1.179	0.985	1.013	1.037	1.175	1.032	1.176	1.037	1.176	1.071	1.201	1.095	0.928
13	2500	0.978	0.982	0.996	1.019	1.024	1.008	0.958	0.979	0.966	0.960	1.046	1.067	1.146	1.085
14	2500	1.008	1.004	0.990	0.985	1.022	0.992	0.984	0.976	0.984	0.961	1.052	1.046	1.104	1.037
15	2500	1.034	1.012	0.982	0.978	1.029	0.996	1.015	0.996	1.018	0.993	1.071	1.043	1.049	0.986
16	2500	1.042	1.023	1.000	0.957	1.037	1.010	1.027	1.013	1.031	1.012	1.072	1.054	1.017	0.964
17	2500	1.020	1.080	0.964	0.992	1.016	1.072	1.009	1.074	1.014	1.075	1.053	1.109	1.016	0.948
18	2500	1.024	1.133	0.962	1.004	1.021	1.129	1.016	1.130	1.021	1.130	1.037	1.150	1.017	0.932
19	500	0.641	0.476	0.963	0.736	1.153	0.913	0.943	0.922	1.098	0.949	1.147	0.846	1.353	0.943
20	500	1.056	0.953	1.038	0.897	1.175	1.016	0.996	0.962	1.043	0.988	1.150	1.007	1.319	1.022
21	500	1.096	1.019	1.054	0.977	1.121	1.019	1.033	0.969	1.072	0.992	1.184	1.059	1.329	1.042
22	500	1.084	1.090	1.023	1.017	1.081	1.060	1.030	1.037	1.060	1.056	1.120	1.118	1.283	1.007
23	500	1.117	1.153	1.018	1.024	1.092	1.117	1.060	1.111	1.086	1.124	1.111	1.166	1.343	0.933
24	500	1.119	1.272	1.037	1.045	1.101	1.248	1.077	1.247	1.100	1.253	1.125	1.256	1.406	0.899
25	500	0.807	0.525	0.893	0.688	1.098	0.867	0.892	0.954	1.079	0.989	1.001	0.863	1.278	0.997
26	500	0.926	0.780	1.016	0.821	1.138	0.937	0.905	0.942	1.029	0.975	1.076	0.956	1.543	1.020
27	500	1.006	1.038	1.027	1.000	1.050	1.015	0.955	0.999	0.992	1.017	1.039	1.090	1.404	1.073
28	500	1.074	1.094	1.013	1.033	1.090	1.050	1.029	1.043	1.057	1.058	1.065	1.118	1.357	1.031
29	500	1.053	1.168	0.998	1.024	1.030	1.124	0.997	1.128	1.023	1.138	1.038	1.174	1.205	0.981
30	500	1.088	1.202	1.016	1.003	1.072	1.180	1.049	1.183	1.071	1.187	1.084	1.189	1.261	0.919
31	500	0.663	0.683	0.759	0.681	1.061	0.797	0.938	0.990	1.228	1.026	1.018	0.875	1.182	0.905
32	500	1.119	0.721	0.914	0.778	1.193	0.831	0.987	0.897	1.182	0.927	1.073	0.890	1.405	1.180
33	500	1.101	0.967	1.067	0.984	1.134	0.932	1.029	0.946	1.091	0.959	1.145	1.035	1.427	1.061
34	500	1.094	1.061	1.032	0.998	1.082	1.006	1.010	1.019	1.048	1.026	1.127	1.077	1.407	0.997
35	500	1.118	1.126	1.063	1.028	1.097	1.084	1.057	1.096	1.087	1.101	1.129	1.117	1.311	0.972
36	500	1.108	1.180	1.064	1.010	1.090	1.158	1.066	1.165	1.091	1.168	1.122	1.154	1.314	0.961

Copyright © 2026 John Wiley & Sons, Ltd.

Supplementary Table 4–2. Ratio of MCSE to MESE for $\mathrm{E}[Y(1)] - \mathrm{E}[Y(0)]$ and $\log(\mathrm{E}[Y(1)]) - \log(\mathrm{E}[Y(0)])$.

Scenario	N	Ratio of MCSE to MESE for													
		$\mathrm{E}[Y(1)] - \mathrm{E}[Y(0)]$							$\log(\mathrm{E}[Y(1)]) - \log(\mathrm{E}[Y(0)])$						
		ML	Unadj	FML	FLIC	FLAC	PS-adj	IPW	ML	Unadj	FML	FLIC	FLAC	PS-adj	IPW
1	2500	0.852	0.979	1.004	0.997	1.150	1.238	0.945	0.984	0.912	0.935	1.096	1.092	1.144	1.093
2	2500	0.962	1.016	0.973	1.027	1.027	1.095	0.923	0.966	0.902	0.932	1.044	1.089	1.221	1.056
3	2500	1.018	1.008	1.021	0.969	1.029	1.024	0.994	0.984	0.994	0.982	1.084	1.088	1.206	1.081
4	2500	0.999	0.991	0.990	0.951	1.002	0.996	0.980	0.972	0.984	0.975	1.061	1.066	1.170	1.103
5	2500	1.005	1.006	0.983	0.979	1.003	1.004	0.992	0.993	0.998	0.998	1.074	1.088	1.205	1.162
6	2500	1.049	1.042	1.024	0.989	1.046	1.039	1.040	1.033	1.045	1.039	1.131	1.138	1.223	1.257
7	2500	0.965	1.081	1.040	0.944	1.228	1.329	0.941	0.966	0.945	0.952	1.082	1.045	1.129	1.084
8	2500	1.010	1.025	1.010	1.055	1.069	1.102	0.974	0.986	0.963	0.967	1.074	1.099	1.201	1.088
9	2500	1.048	1.060	1.045	1.062	1.065	1.080	1.022	1.031	1.023	1.029	1.109	1.128	1.050	1.021
10	2500	1.023	1.029	1.014	1.044	1.025	1.033	1.004	1.010	1.010	1.015	1.086	1.099	1.019	0.973
11	2500	0.997	1.005	0.971	0.986	0.994	1.003	0.985	0.993	0.990	0.999	1.060	1.077	1.123	1.059
12	2500	1.017	1.021	0.990	1.009	1.014	1.018	1.008	1.013	1.013	1.018	1.076	1.091	1.082	1.101
13	2500	1.012	1.038	0.996	1.024	1.096	1.140	0.944	0.941	0.939	0.922	1.058	1.086	1.114	1.140
14	2500	1.008	1.003	0.990	0.991	1.066	1.078	0.969	0.956	0.962	0.940	1.064	1.069	1.082	1.065
15	2500	1.007	0.992	0.979	0.972	1.020	1.010	0.984	0.967	0.985	0.967	1.067	1.058	1.016	0.991
16	2500	1.018	0.994	0.998	0.965	1.021	0.999	1.000	0.976	1.005	0.980	1.070	1.057	0.992	0.957
17	2500	1.008	1.004	0.968	0.982	1.006	1.002	0.996	0.992	1.001	0.997	1.060	1.066	0.993	0.970
18	2500	0.999	1.000	0.965	0.985	0.996	0.997	0.990	0.992	0.995	0.997	1.034	1.047	0.996	0.991
19	500	0.723	0.699	0.966	0.737	1.203	1.187	0.899	0.001	1.051	0.856	1.066	0.919	1.198	0.999
20	500	1.093	1.030	1.036	0.889	1.237	1.253	0.963	0.913	1.012	0.977	1.109	1.037	1.204	1.114
21	500	1.125	1.125	1.057	1.033	1.162	1.183	0.993	0.984	1.031	1.016	1.157	1.128	1.230	1.155
22	500	1.084	1.123	1.029	1.102	1.090	1.116	0.996	1.014	1.030	1.047	1.120	1.153	1.204	1.179
23	500	1.066	1.065	1.024	1.058	1.050	1.048	1.002	1.000	1.034	1.032	1.100	1.112	1.273	1.271
24	500	1.060	1.048	1.032	1.035	1.042	1.031	1.015	1.005	1.040	1.031	1.105	1.092	1.356	1.459
25	500	0.839	0.927	0.885	0.620	1.149	1.144	0.835	0.000	1.006	0.813	0.960	0.862	1.132	0.853
26	500	0.985	1.039	1.009	0.775	1.194	1.211	0.859	0.001	0.970	0.905	1.040	0.952	1.363	0.946
27	500	1.029	1.091	1.021	1.019	1.084	1.129	0.918	0.944	0.955	0.981	1.038	1.089	1.309	1.083
28	500	1.061	1.082	1.008	1.060	1.091	1.102	0.984	0.985	1.017	1.020	1.061	1.108	1.273	1.064
29	500	1.016	1.051	0.986	1.004	1.006	1.037	0.951	0.982	0.981	1.012	1.031	1.076	1.148	1.031
30	500	1.047	1.022	1.006	0.986	1.030	1.007	1.004	0.982	1.029	1.005	1.073	1.063	1.225	1.170
31	500	0.716	0.772	0.756	0.574	1.113	1.132	0.880	0.000	1.152	0.810	1.008	0.863	1.108	0.828
32	500	1.120	0.992	0.911	0.718	1.231	1.187	0.933	0.001	1.121	0.901	1.053	0.928	1.323	0.959
33	500	1.098	1.089	1.065	1.024	1.139	1.148	0.974	0.935	1.038	1.015	1.124	1.091	1.345	1.125
34	500	1.090	1.099	1.031	1.036	1.090	1.093	0.982	0.973	1.024	1.017	1.124	1.126	1.349	1.130
35	500	1.106	1.086	1.067	1.060	1.090	1.071	1.036	1.016	1.071	1.051	1.128	1.111	1.281	1.120
36	500	1.075	1.063	1.050	1.015	1.058	1.047	1.030	1.020	1.057	1.046	1.104	1.103	1.285	1.239

Copyright © **2026** John Wiley & Sons, Ltd.

Prepared using simauth.cls

3. Additional description of the Society of OSSI Study data

Supplementary Table 5. Background risk factors of patients with arthritis by smoking status ($n = 2717$).

		Non-smokers ($n = 2522$)	Smokers ($n = 195$)
Age category	20–29	7(0.26%)	0(0%)
	30–39	15(0.55%)	3(0.11%)
	40–49	68(2.5%)	20(0.74%)
	50–59	235(8.65%)	34(1.25%)
	60–69	585(21.5%)	74(2.72%)
	70–79	1021(37.6%)	48(1.77%)
	80–89	587(21.6%)	16(0.59%)
	90–99	4(0.15%)	0(0%)
	≥ 100	0(0%)	0(0%)
Gender	Male	414(15.2%)	103(3.8%)
	Female	2108(77.6%)	92(3.4%)
ASA classification	≤ 2	2414(88.8%)	189(7%)
	≥ 3	108(4%)	6(0.2%)
Presence or absence of diabetes	Absence	2161(79.5%)	171(6.3%)
	Presence	361(13.3%)	24(0.9%)
BMI category	< 25	1456(53.6%)	111(4.09%)
	25–< 30	796(29.3%)	66(2.43%)
	≥ 30	270(9.94%)	18(0.66%)
Total surgical time	< 60 minutes	53(1.95%)	5(0.18%)
	60–150 minutes	1895(69.8%)	136(5.01%)
	≥ 150 minutes	574(21.1%)	54(1.99%)
Postoperative drainage duration	None	414(15.2%)	29(1.07%)
	< 48 hours	2053(75.6%)	163(6%)
	≥ 48 hours	55(2.02%)	3(0.11%)
Presence of rheumatoid arthritis	No	2303(84.8%)	181(6.7%)
	Yes	219(8.1%)	14(0.5%)
Timing of prophylactic antibiotics	None	3(0.11%)	1(0.04%)
	< 24 hours	1501(55.2%)	135(4.97%)
	≥ 24 hours	1018(37.5%)	59(2.17%)
Highest postoperative blood glucose level	< 200 mg/dL	2047(75.3%)	165(6.07%)
	≥ 200 mg/dL	104(3.83%)	3(0.11%)
	Not measured	371(13.7%)	27(0.99%)
Surgical duration (hours)	Mean	1.6	1.72
	Max	8	5
	Min	0	0
	Median	1	2
Blood loss (ml)	Mean	302.6	236.3
	Max	3100	1300
	Min	0	0
	Median	200	116
Incidence of SSI within 30 days after surgery	No occurrence	2510(92.4%)	192(7.1%)
	Occurrence	12(0.4%)	3(0.1%)

 Copyright © 2026 John Wiley & Sons, Ltd.

Statistics in Medicine

4. R code for simulation experiments

Note. For the complete simulation code and input data (true value CSV files), please refer to our GitHub repository: github.com/shashibe/statmed.

Copyright © 2026 John Wiley & Sons, Ltd.

Prepared using simauth.cls
